# Genome-wide CRISPR screen identifies host dependency factors for influenza A virus infection

**DOI:** 10.1038/s41467-019-13965-x

**Published:** 2020-01-09

**Authors:** Bo Li, Sara M. Clohisey, Bing Shao Chia, Bo Wang, Ang Cui, Thomas Eisenhaure, Lawrence D. Schweitzer, Paul Hoover, Nicholas J. Parkinson, Aharon Nachshon, Nikki Smith, Tim Regan, David Farr, Michael U. Gutmann, Syed Irfan Bukhari, Andrew Law, Maya Sangesland, Irit Gat-Viks, Paul Digard, Shobha Vasudevan, Daniel Lingwood, David H. Dockrell, John G. Doench, J. Kenneth Baillie, Nir Hacohen

**Affiliations:** 1 Harvard University Virology Program, Harvfvard Medical School, Boston, MA02142 USA; 2 Broad Institute of MIT and Harvard, 415 Main Street, Cambridge, MA 02142 USA; 3 Roslin Institute, University of Edinburgh, Easter Bush, EH25 9RG UK; 4 Harvard-MIT Health Sciences and Technology, Harvard Medical School, Boston, MA 02115 USA; 5 School of Molecular Cell Biology and Biotechnology, Department of Cell Research and Immunology, George S. Wise Faculty of Life Sciences, Tel Aviv University, Tel Aviv, Israel; 6 School of informatics, University of Edinburgh, Edinburgh, EH8 9YL UK; 7 Center for Cancer Research, Massachusetts General hospital, Harvard Medical School, Boston, MA USA; 8 The Ragon Institute of Massachusetts General Hospital, MIT and Harvard University, Cambridge, MA USA; 9 MRC Center for Inflammation Research, University of Edinburgh, Edinburgh, UK; 10 Intensive Care Unit, Royal Infirmary Edinburgh, Edinburgh, EH16 5SA UK; 11 Massachusetts General Hospital Cancer Center, Boston, MA 02129 USA

**Keywords:** CRISPR-Cas9 genome editing, Influenza virus

## Abstract

Host dependency factors that are required for influenza A virus infection may serve as therapeutic targets as the virus is less likely to bypass them under drug-mediated selection pressure. Previous attempts to identify host factors have produced largely divergent results, with few overlapping hits across different studies. Here, we perform a genome-wide CRISPR/Cas9 screen and devise a new approach, meta-analysis by information content (MAIC) to systematically combine our results with prior evidence for influenza host factors. MAIC out-performs other meta-analysis methods when using our CRISPR screen as validation data. We validate the host factors, *WDR7, CCDC115* and *TMEM199*, demonstrating that these genes are essential for viral entry and regulation of V-type ATPase assembly. We also find that *CMTR1*, a human mRNA cap methyltransferase, is required for efficient viral cap snatching and regulation of a cell autonomous immune response, and provides synergistic protection with the influenza endonuclease inhibitor Xofluza.

## Abstract (web summary)

Here, Li et al. perform a genome-wide CRISPR screen to identify host dependency factors for influenza A virus infection and show that the host mRNA cap methyltransferase CMTR1 is important for viral cap snatching and that it affects expression of antiviral genes.

## Introduction

Influenza A Virus (IAV) causes acute respiratory infections in humans and poses a major threat to public health and the global economy. The 2009 H1N1 pandemic resulted in over 60 million infected cases in the United States^1^ and more than 120,000 deaths worldwide, the majority of which were in young people (<65 years old)^2^. Avian influenza strains like the H5N1 and H7N9 have also crossed the species barrier and caused lethal infections in humans in recent years^3–5^, raising concerns for future pandemics. Although vaccination against seasonal influenza is an essential part of the public health strategy, its efficacy is variable, and there are few therapeutic options for people who become infected. Conventional antiviral therapies including neuraminidase inhibitors (e.g., oseltamivir, zanamivir) and M2 channel blockers (e.g., amantadine) have limited efficacy and are vulnerable to the rapid selection of resistant virus in treated patients^6–8^. A new class of endonuclease inhibitor (Xofluza) has been approved recently^9^, but faces similar issues with emergence of resistance viral strains^10^.

Like most viruses, IAV has a relatively small genome and limited repertoire of encoded proteins^11^ and relies on the host machinery to replicate and complete its life cycle. Identification of host dependency factors (HDFs) that are necessary for IAV replication thus provides an attractive strategy for discovering new therapeutic targets, since the evolution of resistance to host-targeted therapeutics is expected to be slower^12–14^. To achieve this end, numerous large-scale RNA interference (RNAi) screens have been performed in the past, reporting a total of 1362 HDFs that are important for IAV replication^15–21^. While these screens provided valuable insights into viral-host interactions^22–24^, overlap in the identified hits has been limited^25^, a result that likely stemmed from differences in experimental conditions as well as intrinsic limitations in the RNAi technology. A similar inconsistency is evident among screens for HDFs required for HIV infection^26–28^.

In recent years, many groups have successfully utilized CRISPR/Cas9 as an alternative screening strategy for HDFs in viral infections^29–33^. A recently published genome-wide CRISPR/Cas9 screen based on cell survival after IAV infection uncovered a number of new HDFs involved in early IAV infection, but shared few hits with previous RNAi screens. This raises the question whether CRISPR- and RNAi-based screens are confined to identifying mutually exclusive targets due to technological biases.

To more comprehensively identify IAV-host interactions, we perform pooled genome-wide CRISPR/Cas9 screens and use IAV hemagglutinin (HA) protein expression on the cell surface as a phenotypic readout. We identify an extensive list of IAV HDFs, including new and previously known factors, involved in various stages of the IAV life cycle. We focus on the less understood host factors and discover that loss of *WDR7, CCDC115*, and *TMEM199* results in lysosomal biogenesis and over-acidification of the endo-lysosomal compartments, which blocks IAV entry and increases degradation of incoming virions. We also identify the human 2′O-ribose cap methyltransferase, *CMTR1* as an important host factor for IAV cap snatching and regulator of cell autonomous immune surveillance. To link our findings to previously identified IAV HDFs, we devise a new approach, meta-analysis by information content (MAIC), to combine data from diverse sources of unknown quality, in the form of ranked and unranked gene lists. MAIC performs better than other algorithms for both synthetic data and in an experimental test, and provides a comprehensive ranked list of host genes necessary for IAV infection.

## Results

### Influenza host dependency factors identified in a CRISPR screen

**Fig. 1:**
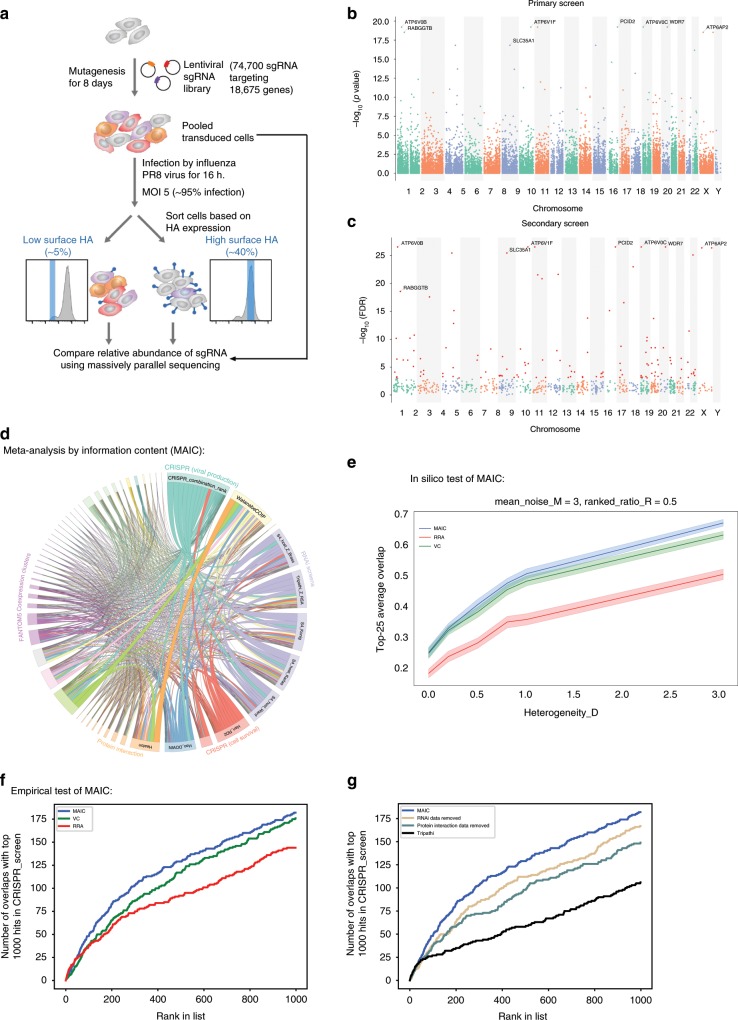
Genome-wide CRISPR screens identify IAV host dependency factors. **a** Schematics of the genome-wide CRISPR/Cas9 screening strategy. **b**, **c** Spatial distribution of CRISPR knockout signals on the genome for the primary and secondary screens. **b** Gene-level *p*-values in primary screen. A selection of top hits is highlighted. **c** Gene-level FDRs for top hits in the secondary screen. The core set of 121 robust hits (FDR < 0.05) is shown highlighted in red. **d** Representation of shared information content between each data source after MAIC analysis. Size of data source blocks is proportional to the summed information content (MAIC scores) of input list. Lines are colored according to the dominant data source. Data source categories share the same color; the largest categories and data sources are labeled (see supplementary information for full source data). CRISPR screen performed in this study is labeled as CRISPR (viral production). **e** Example of validation of MAIC screen using synthetic data (see methods). Y-axes show overlap ratio compared to a known truth (see methods; narrow line = average of 100 replicates; shading = 95% confidence interval). MAIC consistently out-performs existing methods (robust rank aggregation, RRA; vote counting; VC) when presented realistic inputs. Example shown is for a mixture of ranked and unranked data sources, and moderate variation in data quality (heterogeneity) between data sources. See supplementary Figs. 2 for full evaluation. **f** Experimental validation of MAIC (computed without CRISPR data) against an unseen gold standard (CRISPR screen). Plot shows number of overlaps with the top 1000 hits in CRISPR screen for a given position in each ranked dataset. **g** Contribution of different data categories to predictive value of the MAIC. As in (**f**), graph shows number of overlaps with the top 1000 CRISPR screen hits. Results are shown with all RNAi data from previous studies removed from the input set, and with all proteomic or protein interaction data removed. The results of the systematic meta-analysis by Tripathi et al.^36^ are shown for comparison.

To identify HDFs that are necessary for IAV infection, we performed two independent rounds of pooled genome-wide CRISPR screens in A549-Cas9 cells using the well-established AVANA4 lentivirus library^34^, which encodes 74,700 sgRNAs targeting 18,675 annotated protein-coding genes (with 4 sgRNAs per gene), as well as 1000 non-targeting sgRNAs as controls. On day 9 post-transduction with the library, we infected ~300 million puromycin-resistant cells with influenza A/Puerto Rico/8/1934 (PR8) virus at multiplicity of infection (MOI) 5 for 16 h. Cells were sorted by FACS into different bins based on their levels of surface viral HA (Fig. 1a), which should reflect the efficiency of the viral life cycle from entry to HA export. Roughly ~5% of the cells were sorted into the uninfected bin (low HA expression); these were compared to a control population of cells (comprising the mode for HA expression +/− 20% of the population). Cells that harbor genetic alterations restricting influenza virus replication (i.e., sgRNAs that target host genes important for infection) are expected to be enriched in the uninfected bin. For analysis of the screen data, we combined the empirical *p*-values which sums the evidence in support of over-representation of sgRNAs targeting a given gene. This method optimizes the discovery power of the screen and is more reproducible than two other common analysis approaches—STARS and MAGeCK (Supplementary Fig. 1, Supplementary Note 1). In this initial screen, sgRNAs targeting 41 genes were significantly enriched in the uninfected bin relative to control bin (FDR < 0.05) (Supplementary Data 1, Fig. 1b).

**Fig. 2:**
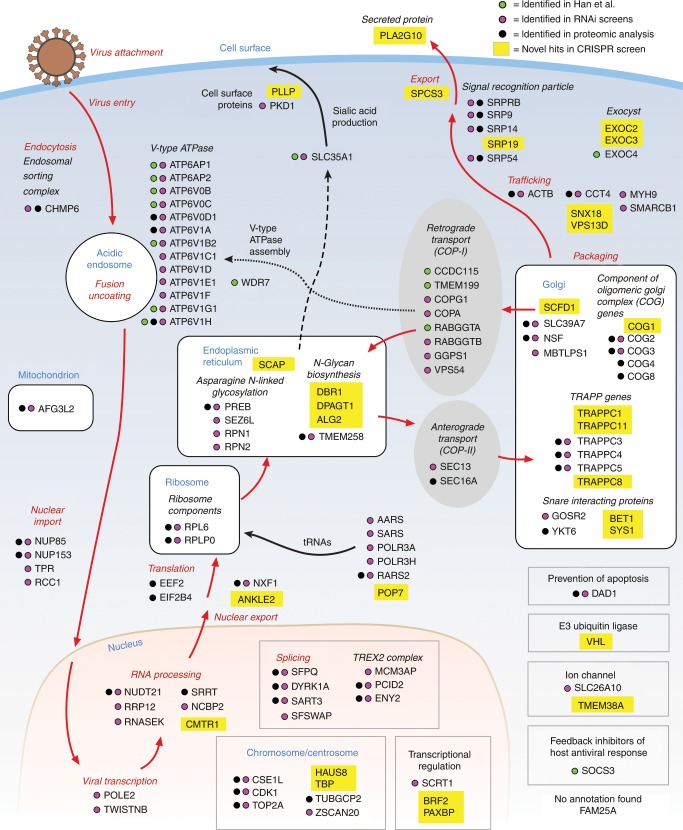
CRISPR screens hits and their predicted roles in the IAV life cycle. Diagram showing all 121 hits from our CRISPR screen (FDR < 0.05) and their predicted roles in the IAV life cycle. Hits are marked by a colored dot if they had also been identified in previous RNAi screens (Magenta), proteomic studies (Black) or CRISPR screen (Green). Hits unique to our CRISPR screen are highlighted in yellow.

To validate identified hits (and thus reduce false positives) and recover additional hits (minimize false negatives), we performed a secondary pooled screen targeting the top 1000 ranked hits from the primary screens but with 10 sgRNAs per gene. We re-identified 37 out of the 41 hits that scored with FDR < 0.05 in the primary screen, as well as recovering additional hits that failed to meet the original FDR cutoff (Fig. 1c). Combining data from the primary and secondary screens yielded a final list of 121 genes (FDR < 0.05) whose roles have been shown or predicted in different stages of the IAV life cycle (Fig. 2, Supplementary Data 1). Amongst these, 78 genes showed significant enrichment of two or more sgRNAs in the uninfected bin, while 43 genes had enrichment for only a single sgRNA. We included the latter in our analysis in order to maximize the discovery power for subsequent validation, and because many of these genes have also been identified in previous RNAi screens and proteomics studies.

The significantly-enriched genes from the primary and secondary screens included both known HDFs from previous RNAi screens such as ATPase subunits, components of the vesicular transport pathway, signal recognition particles, and genes involved in sialic acid synthesis, as well as unknown ones like components of the TRAPP and TREX2 complexes, genes involved in protein prenylation and co-factors of V-type ATPases. Unlike the previous RNAi screens, we found relatively few ribosomal subunits and genes involved in translation and splicing among our top ranked hits, suggesting that CRISPR-mediated editing of essential host factors potently reduces cell survival, such that cells bearing these edits did not survive the 8 days between editing and influenza virus challenge.

### Meta-analysis by information content (MAIC)

To incorporate these findings into the existing evidence base, which include annotated pathways^23,35^, genetic perturbation screens^16,33,36^, and protein–protein interactions, we devised the MAIC approach to evaluate the information content in each data source by comparing it to other data sources. MAIC takes a simple and intuitive approach to quantify the information content in a given list of genes, for example the results of a single experiment, by comparing it to the results of other experiments that might reasonably be expected to find some of the same genes. In this way MAIC produces a weighting factor for each experiment, and then calculates a score for each gene. Our analysis then produced a final ranked list of HDFs based on this score, which summarizes the composite evidence from all input sources of a particular gene being involved in IAV infection (Supplementary Data 2). We found that our CRISPR/Cas9 screen provides the most information (11.4% of total information content) when compared with individual genetic perturbation screens and proteomics studies performed in the past (Fig. 1d).

We performed extensive in silico validation of the MAIC method using synthetic data designed to test MAIC when presented with combinations of ranked and unranked data, varying levels of noise, and varying levels of heterogeneity of data quality in the input data sets. We compared MAIC to two existing approaches: (1) a simple count of the number of occurrences of each gene in each data set, and (2) robust rank aggregation (RRA), a powerful method for aggregating ranked data (such as screen results), which does not allow for the inclusion of unranked data (such as a pathway or coexpression cluster)^37^. MAIC performs better than both methods under most conditions; in the absence of noise (when every single item in the input dataset is correct), MAIC performs similarly to other methods (Fig. 1e, Supplementary Fig. 2).

In order to provide an experimental test of the MAIC algorithm, we used MAIC to combine relevant data sources from the literature (Supplementary Note 2), with the exception of the new data from our CRISPR screen. We then used the CRISPR screen as an unseen “gold standard”, against which to test other siRNA screens and meta-analysis. Both MAIC and RRA successfully prioritize highly-ranked genes in the top 50 CRISPR hits, but RRA fails to identify hits below this level (Fig. 1f). In contrast, a simple count of the occurrences of each gene in each category (vote counting) fails to prioritize the top candidates, but is more effective at identifying many genes in the top 1000 ranks. This is in part due to the dominance of protein interaction data in the MAIC results (Fig. 1g). The MAIC algorithm outperforms both RRA and vote-counting, and prioritizes more genes that overlap with CRISPR results than any other data source (RNAi screens and protein interaction studies), including a previous gene-level meta-analysis^36^ (Fig. 1g). MAIC thus identifies a unique set of host factors based on multiple lines of evidence, and distinct from the ranked list of any individual screen (Supplementary Data 2). Ribosomal genes feature heavily in the MAIC results because of strong support from several datasets. As expected, genes with a variety of other functions, including host antiviral response, RNA processing and proteasome function are also highly supported. Gene set enrichment analysis (GSEA) highlights afferent signaling pathways, including Toll-like receptor signaling (KEGG; Supplementary Data 2), and *EGF* and *MAPK* signaling and related pathways (BioCarta; Supplementary Data 2).

### Validation of influenza host factor dependencies

We selected 28 genes for further validation based on their top ranking in our screen and not being previously implicated in IAV infection. A549 cells were transduced with the top 2 sgRNAs from the secondary screen (based on fold change of sgRNA in uninfected bin relative to control bin) and genome editing was confirmed by sequencing of the predicted target sites. Polyclonal KO cells were then infected with Influenza A PR8 virus at MOI 5 on day 9 post-sgRNA transduction and stained for surface HA. We found 21 out of the 28 polyclonal KO cell lines to be partially protected against IAV infection for both sgRNAs (Supplementary Fig. 3), while three polyclonal KO cell lines were protected for only one of the two tested sgRNAs. The degree of protection varied between the cell lines despite their sgRNAs having comparable genome editing efficiency (Supplementary Fig. 4), suggesting the roles of these genes differ depending on the cell context.

**Fig. 3:**
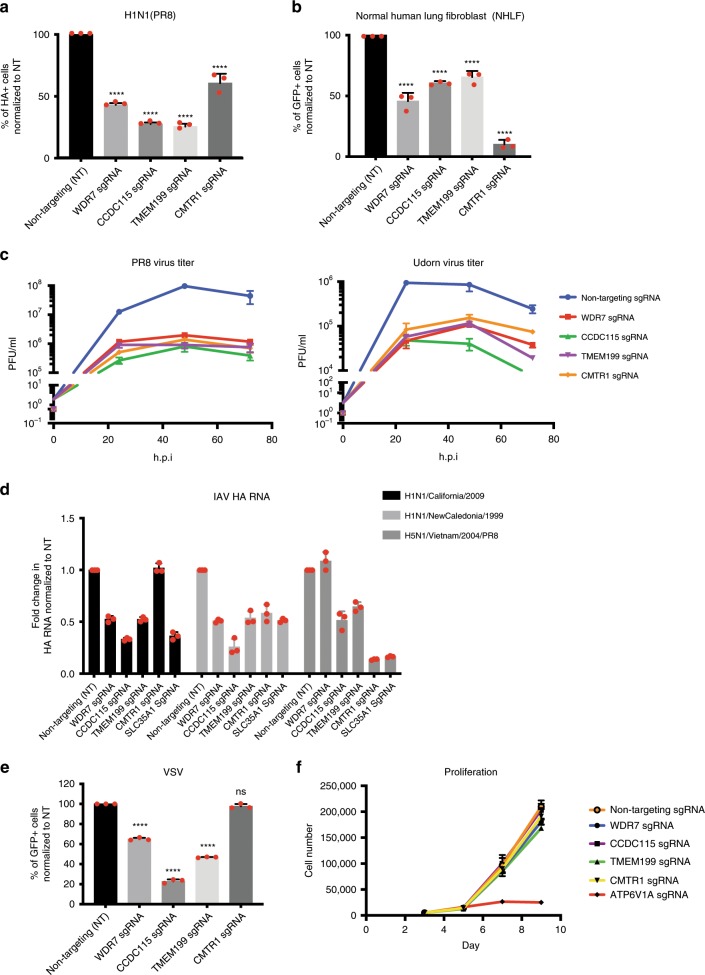
Validation of screen hits in A549 cells and normal human lung fibroblasts (NHLF). **a** A549 cells and **b** primary normal human lung fibroblasts were transduced with either gene-specific or non-targeting sgRNA, followed by infection with PR8 virus at MOI 5 for 16 h. *Y*-axis shows percentage of HA-positive cells normalized to non-targeting sgRNA. Error bars represent standard deviations from three independent experimental replicates. **c** Virus titer in plaque forming units (PFU)/ml at 24, 48, and 72 h post-infection with either PR8 (Left) or Udorn (right) virus at MOI 0.1. Supernatant used for the plaque assay was collected from infected A549 cells transduced with either gene-specific or non-targeting sgRNA. Error bars represent standard deviations from three independent experimental replicates. **d** Fold change in viral HA RNA level relative to GAPDH measured by qRT-PCR. A549 cells transduced with either gene-specific or non-targeting sgRNA were infected with H1N1 California/2009, H1N1/New Caledonia/1999 or H5N1/Vietnam/2004/PR8-recombinant IAV strains at high MOI for 16 h. Fold change was normalized to HA RNA level in A549 cells transduced with non-targeting sgRNA. A549 cells transduced with SLC35A1 sgRNA was used for comparison. Error bars represent standard deviations from three independent experimental replicates. **e** A549 cells transduced with either gene-specific or non-targeting sgRNA were infected with VSV-GFP virus at MOI 1 for 16 h. *Y*-axis shows percentage of GFP-positive cells normalized to non-targeting sgRNA. Error bars represent standard deviations from three independent experimental replicates. **f** Proliferation curve of transduced A549 cells up to 9 days post sgRNA-transduction. Error bars represent standard deviations from three independent experimental replicates. ****P = 0.0001 ***P < 0.001 and *P < 0.05, by one-way ANOVA test.

Deletion of four of the hits—*WDR7, CCDC115*, *TMEM199*, and *CMTR1*—conferred strong protection against PR8 virus infection in both A549 cells and normal human lung fibroblasts (NHLFs) (>40% reduction in percentage of HA-positive cells) (Fig. 3a, b). To test if the four genes are required for efficient virus production, we infected WDR7, CCDC115, TMEM199, and CMTR1 polyclonal KO cells with H1N1 PR8 virus and H3N2 Udorn virus at MOI 0.1 and monitored virus production at 24, 48, and 72 h post-infection by plaque assay. Virus production peaked after 48 hours post-infection for PR8 virus and 24 h post-infection for Udorn virus. At these time points, we observed >2 log reduction in virus titer in all four polyclonal KO cell lines for PR8 virus and >1 log reduction in for Udorn virus compared to wild type cells (Fig. 3c). The greater magnitude of reduction in viral infection rate observed at low MOI is likely due to cumulative effects of multiple replication cycles. We also compared the phenotype of knocking out WDR7, CCDC115, TMEM199, and CMTR1 to SLC35A1, a known IAV host factor that was both highly-ranked in ours and previous published CRISPR/Cas9 screens^33^. SLC35A1 is a CMP-sialic acid transporter that is required for surface sialic acid expression and IAV entry. We observed similar reduction in percentage of HA-positive cells and virus titer produced by infected WDR7, CCDC115, TMEM199, and CMTR1 KO cells compared to SLC35A1 KO cells (Supplementary Fig. 5A-E). The degree of protection conferred by CRISPR deletion of these genes is also consistent with what is previously published for polyclonal SLC35A1 KO cells^33^, suggesting that these genes may indeed serve as important IAV HDFs.

Since PR8 and Udorn are lab-adapted IAV strains, we also tested the infectivity of WDR7, CCDC115, TMEM199, and CMTR1 polyclonal KO cells by more recent clinical isolates of IAV including the 1999 New Caledonia and 2009 California H1N1 pandemic strains, as well as a H5N1 IAV strain (Vietnam/2004) that has been re-engineered with PR8 internal genes. Similar to previous observation with PR8 and Udorn virus, we showed that A549 cells lacking WDR7, CCDC115, TMEM199 or CMTR1 again displayed lower levels of IAV HA RNA at 16 h post-infection compared to control cells by qRT-PCR (Fig. 3d).

To confirm that the observed phenotype is not due to off-target effects, we expressed codon-mutated versions of these genes in the polyclonal KO cells and observed restoration of normal IAV infection levels (Supplementary Fig. 6A, Supplementary Fig. 6B) in all the KO cells with the exception of WDR7 KO cells, which is only partially rescued. We speculated that this could be due to the large protein size of WDR7 which makes it difficult to express (173kDA). To confirm that the phenotype type observed for WDR7 is not due to off-target effects, we tested two additional sgRNAs against WDR7 and observed similar reduction in IAV infection rate (Supplementary Fig. 6C). To test if the functions of these genes apply to other viruses, we infected the four polyclonal KO cell lines with vesicular stomatitis virus (VSV) and showed that WDR7, CCDC115, and TMEM199, but not CMTR1 were also required for efficient VSV infection (Fig. 3e).

To test if these genes are essential for cell survival, we monitored the proliferation rate of A549 cells up to 9 days post-transduction with WDR7, CCDC115, TMEM199, and CMTR1 sgRNAs. We observed no significant difference in proliferation rate between these cells compared to those transduced with non-targeting sgRNA (Fig. 3f). In contrast, majority of the cells transduced with sgRNA against ATP6V1A, a V-type ATPase subunit and a known IAV host factor, died by day 7 post-transduction. Annexin V straining also confirmed a similar number of live cells between A549 cells transduced with WDR7, CCDC115, TMEM199, and CMTR1 sgRNAs and non-targeting sgRNA on day 9 post-transduction (Supplementary Fig. 6D). Thus, the four identified genes were critical for IAV infection but not observed to impact cell viability.

### WDR7, CCDC115, TMEM199, and CMTR1 involved in early infection

**Fig. 4:**
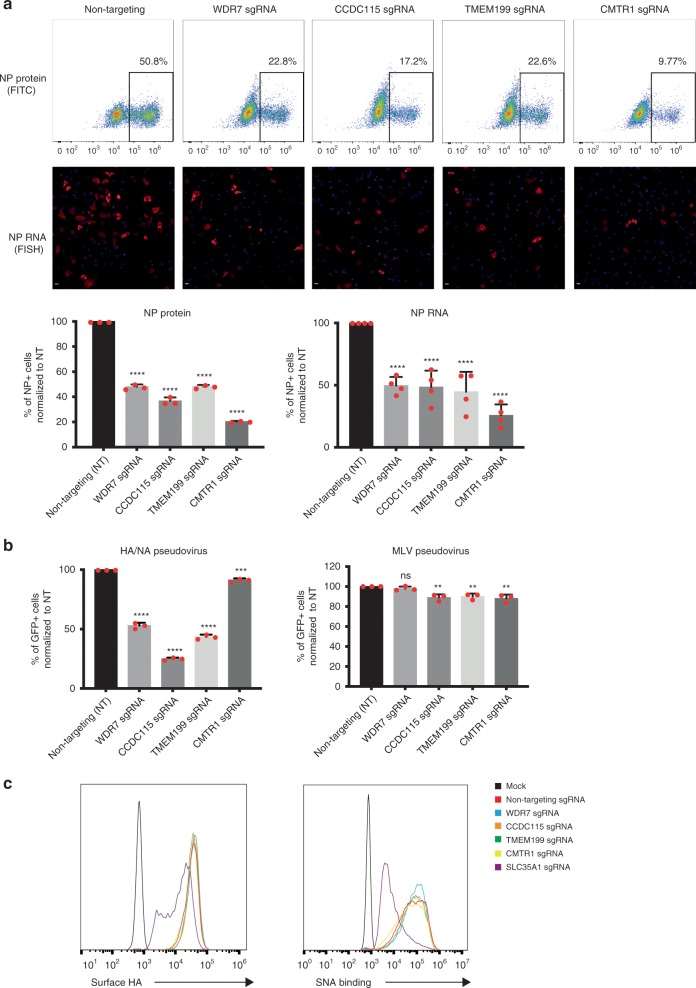
WDR7, CCDC115, TMEM199 and CMTR1 are involved in early infection. **a** Flow cytometry and fluorescent in situ hybridization (FISH) for influenza PR8 NP protein and RNA. A549 cells were transduced with either gene-specific or non-targeting sgRNA and infected with PR8 virus for 4 h. Infected cells were stained with anti-H1N1 NP antibody or NP FISH RNA probes. Bar graphs show quantification for the percentage of NP-positive cells for both protein and RNA (cells with positive FISH staining) normalized to non-targeting sgRNA. Scale bar = 20 μm. Error bars represent standard deviation from three independent experimental replicates for NP protein staining and four randomly chosen frames for NP RNA FISH. **b** A549 cells were transduced with either gene-specific or non-targeting sgRNA and infected with MLV-GFP reporter virus pseudo-typed with either influenza PR8 HA and NA or MLV envelop proteins. Y-axis shows percentage of GFP-positive cells normalized to non-targeting sgRNA. Error bars represent standard deviations from three independent experimental replicates. **c** Histogram showing level of surface-bound HA (left) and sialic acid (right). A549 cells transduced with either gene-specific or non-targeting sgRNA were incubated with PR8 virus for 30 min at 4 °C for monitoring level of surface-bound HA or stained with fluorescein-labeled Sambucus Nigra Lectin for 1 h at 4 °C for surface sialic acid. Cells transduced with sgRNA targeting SLC35A1 was used as positive control. Histograms shown is representative of three independent experimental replicates. ****P = 0.0001 ***P < 0.001 and **P < 0.01, by one-way ANOVA test.

To better understand how loss of these genes conferred resistance against IAV infection, we first determined which steps of the IAV life cycle they play a role in. We found significant reduction in viral nucleoprotein (NP) RNA and protein levels at 4 h post-infection in WDR7, CCDC115, TMEM199, and CMTR1 polyclonal KO cells compared to wild type cells, suggesting that all 4 genes are important during early infection (Fig. 4a)^38^. To test if the genes are required for IAV entry, we infected polyclonal KO cells with MLV-GFP retrovirus pseudotyped with H1N1 PR8 HA and NA proteins. This allows the retrovirus to enter the cell in a HA dependent manner that is akin to IAV entry^39^. We then monitored GFP expression in the cells 48 h post-infection. We found that WDR7, CCDC115 and TMEM199 KO cells, but not CMTR1 KO cells had lower percentage of GFP-expressing cells compared to wild type (Fig. 4b). In contrast, all four polyclonal KO cell lines had comparable GFP expression to wild type cells when infected with an MLV-GFP retrovirus pseudotyped with amphotropic MLV-envelope protein, suggesting that WDR7, CCDC115 and TMEM199 are specifically required for IAV entry in a HA/NA dependent manner. We next asked if the three genes were required for IAV entry by allowing virus attachment to the cell surface membrane. To test this, we incubated polyclonal KO cells with PR8 virus at 4 °C for 30 min (to prevent viral fusion), followed by washing and staining for surface bound HA. We found no difference in HA staining between the KO and wild type cells, suggesting that the genes are not essential for virus attachment (Fig. 4c). This is also supported by the observation that WDR7, CCDC115, and TMEM199 did not affect expression of cell surface sialic acids (Fig. 4c), which serve as entry receptors for IAV^40^. In contrast, A549 cells that have undergone CRISPR deletion of SLC35A1 have both reduced levels of surface sialic acid and bound virions.

### WDR7, CCDC115, and TMEM199 regulate endo-lysosomal pH

**Fig. 5:**
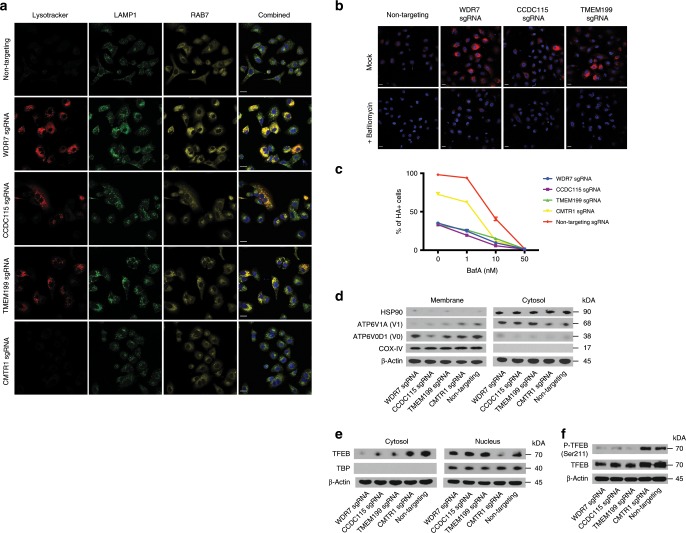
WDR7, CCDC115 and TMEM199 regulate endo-lysosomal pH. **a** Immunofluorescence of A549 cells transduced with either gene-specific or non-targeting sgRNA and stained with Lysotracker red, anti-LAMP1 and anti-Rab7 antibodies. Scale bar = 20 μm. **b** Lysotracker red staining of A549 cells transduced with either gene-specific or non-targeting sgRNA. Cells were mock-treated or treated with 100 nM Bafilomycin A (BafA). Scale bar = 20 μm. **c** A549 cells transduced with either gene-specific or non-targeting sgRNA were treated with different concentrations of BafA followed by PR8 virus infection. Error bars represent standard deviations from two independent experimental replicates. **d** A549 cells transduced with either gene-specific or non-targeting sgRNA were fractioned into membrane and cytosolic components. The two fractions were then subjected to SDS-PAGE and Western blotting using antibodies against subunit A of the V_1_ domain and subunit D of the V_0_ domain as measure of V-ATPase assembly. Western blotting was also performed for HSP90 (cytosolic) and COX-IV (membrane) as positive controls. **e** Cytosolic and nuclear proteins were extracted from A549 cells transduced with either gene-specific or non-targeting sgRNA using the NE-PER Nuclear and Cytoplasmic extraction kit. Samples in each fraction were subjected to SDS-PAGE and western blotting using antibodies against TFEB. Western blotting for TATA-binding protein (TBP) was used as positive control. **f** Whole cell lysates were extracted from A549 cells transduced with either gene-specific or non-targeting sgRNA. Samples were subjected to SDS-PAGE and western blotting using antibodies against TFEB and Phospho-TFEB (ser211).

Recent studies have reported WDR7, CCDC115, and TMEM199 as factors associated with mammalian V-type ATPases^41,42^, but their functions remain unclear. To test if these genes are required for IAV entry by regulating endo-lysosomal acidification, we stained WDR7, CCDC115, TMEM199, and CMTR1 polyclonal KO cells with lysotracker red, fluorescent-labeled anti-Rab7 and anti-LAMP1 antibodies. Unexpectedly, we observed an increase in lysotracker red staining in WDR7, CCDC115, and TMEM199 KO cells, which co-stained partly with Rab7 (late endosome) and LAMP1 (lysosome). (Fig. 5a). To determine if the increase in lysotracker red staining is solely due to expansion of the endo-lysosomal compartments or actual reduction in pH, we also stained the cells with the more pH-sensitive lysosensor blue dye and Oregon Green Dextran. As in the case with lysotracker red, we observed an increase in lysosensor blue staining and reduction in Oregon Green signal (Oregon Green fluorescence becomes quenched at lower pH) in WDR7, CCDC115 and TMEM199 KO cells, indicating that both endo-lysosomal expansion and reduction in pH were taking place (Supplementary Fig. 7A). A similar increase in lysotracker staining is observed in NHLFs transduced with WDR7, CCDC115, and TMEM199 sgRNAs (Supplementary Fig. 7B). We next asked if this reduction in endo-lysosomal pH could be restored in WDR7, CCDC115, and TMEM199 polyclonal KO cells by treating the cells with Bafilomycin A (BafA), a known inhibitor of V-type ATPase activity^43^ and IAV infection^44^. We observed a reduction in lysotracker staining in KO cells treated with BafA treatment (Fig. 5b), suggesting that these genes function upstream of V-type ATPases. However, we found that BafA treatment, even at low concentrations, further protected the KO cells against IAV infection (Fig. 5c, Supplementary Fig. 8A). We speculated that this may be due to disruption of the fine pH gradient in the endocytic pathway that is required for efficient IAV uncoating and replication^45^. WDR7, CCDC115 and TMEM199 appeared to play non-redundant roles as over-expression of WDR7 in CCDC115 or TMEM199 polyclonal KO cells and vice versa did not rescue IAV infection rate (Supplementary Fig. 8B). Over-expression of WDR7, CCDC115 and TMEM199 in wild type A549 cells also did not have an effect on lysotracker staining or IAV infectivity, suggesting that their effects on function may already be saturated at the steady state (Supplementary Fig. 8C, Supplementary Fig. 8D).

To understand how loss of WDR7, CCDC115, and TMEM199 resulted in expansion and over-acidification of the endo-lysosomal compartments, we extracted cytosolic and membranous proteins from the polyclonal KO cells and measured the relative abundance of the cytosolic V_1_A and transmembrane V_0_D domain subunits of the V-type ATPases via western blot^46^. We observed an enrichment of V_1_A subunit in the cytosolic fraction of WDR7, CCDC115 and TMEM199 KO cells and a corresponding reduction in the membrane fraction, indicating that at least a subset of V-type ATPases are in a dis-assembled and less active state in these cells (Fig. 5d). This was unexpected as inactivation of the V-type ATPase should in theory lead to less endo-lysosomal acidification. It has been reported that prolonged treatment of cells with lysosomotropic compounds such as chloroquine and tamoxifen could lead to increased lysotracker red staining due to lysosome adaptation and biogenesis caused by nuclear translocation of transcription factor EB (TFEB)^47,48^ (Supplementary Fig. 9A). To test if absence of WDR7, CCDC115 or TMEM199 leads to TFEB translocation and lysosomal biogenesis, we extracted cytosolic and nuclear proteins from WDR7, CCDC115, and TMEM199 polyclonal KO cells and measured the relative abundance of TFEB in each fraction. We observed an enrichment of TFEB in the nuclear fraction of WDR7, CCDC115 and TMEM199 KO cells but not in CMTR1 KO or wild-type cells (Fig. 5e). There was also de-phosphorylation of TFEB at Ser211 in WDR7, CCDC115, and TMEM199 KO cells, which is required for TFEB dissociation from the lysosomal surface and subsequent nuclear translocation^49^ (Fig. 5f). Sequencing of bulk RNA from the KO cells also showed an increase in expression of lysosomal genes including ASAH1, NPC2, Cathepsin B, and Cathepsin L (Supplementary Fig. 9B)^50^. These led us to conclude that the loss of WDR7, CCDC115, and TMEM199 results in V-type ATPase inactivation which in turn triggers compensatory lysosomal adaptation and biogenesis. Since Bafilomycin A treatment reduced lysotracker red staining in WDR7, CCDC115 and TMEM199 KO cells, we speculated that different isoforms of V-type ATPase or other ATPases (P- or F-type) may play a compensatory role in these cells when one or more V-type ATPases become inactivated.

### Loss of WDR7, CCDC115 or TMEM199 prevents IAV nuclear entry

**Fig. 6:**
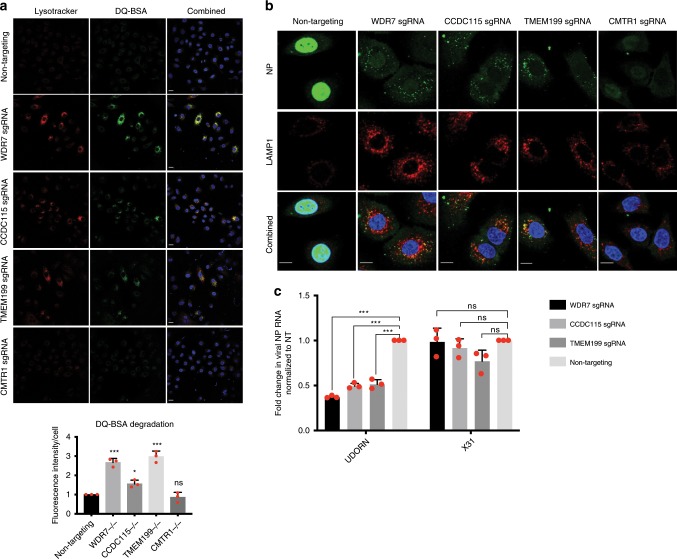
Loss of WDR7, CCDC115 and TMEM199 prevents IAV nuclear entry. **a** Immunofluorescence staining of A549 cells transduced with either gene-specific or non-targeting sgRNA with DQ-green bovine serum albumin (BSA) and Lysotracker red. Cells were treated with 20ug/ml DQ-BSA and Lysotracker red for 1 h at 37 °C followed by fixation to monitor uptake and hydrolysis of the DQ-BSA. DQ-BSA staining was quantified by dividing total fluorescence intensity by the number of cells in each frame. Bar graph shows the relative fluorescence intensity between different sgRNAs. Scale bar = 20 μm. Error bars represent standard deviation from three randomly chosen frames. **b** Immunofluorescent staining of A549 cells transduced with either gene-specific or non-targeting sgRNA with FITC-conjugated anti-H1N1 NP antibody. Cells were infected with PR8 virus at MOI 500 for 2 h at 37 °C. Scale bar = 15 μm. **c** Fold change in viral NP RNA level relative to GAPDH measured by qRT-PCR. A549 cells transduced with gene-specific or non-targeting sgRNA were infected with either influenza H3N2 Udorn or X31 virus at MOI 5 for 16 h. Fold change was normalized to viral NP RNA level in A549 cells transduced with non-targeting sgRNA. Error bars represent standard deviations from three independent experimental replicates. ***P < 0.001 and *P < 0.05, by one-way ANOVA test.

While it is known that an acidic endo-lysosomal environment is required for IAV entry^51^, we showed that expansion of the endo-lysosomal compartment and reduction in pH also block IAV infection in WDR7, CCDC115, and TMEM199 KO cells. To assess the functional effect of lysosomal adaptation, we incubated WDR7, CCDC115 and TMEM199 polyclonal KO cells with DQ-Green BSA, a derivative of bovine serum albumin (BSA) that is heavily labeled with green fluorescent BODIPY FL dye. The dye is usually self-quenched but produces a bright fluorescence when DQ-Green BSA is cleaved by hydrolases in the acidic endo-lysosomal compartments^52^. We found that WDR7, CCDC115, and TMEM199 polyclonal KO cells exhibited brighter DQ-BSA staining than CMTR1 KO cells and wild type cells, which co-stained with the increased lysotracker red signal. This suggested that there is increased endo-lysosomal trafficking and degradation of incoming endocytic cargo in cells lacking WDR7, CCDC115 or TMEM199 (Fig. 6a).

To test if incoming IAV virions are being trafficked to and degraded in the endo-lysosome compartments, we infected polyclonal KO cells with PR8 virus at MOI 500 and stained for intracellular NP protein at 2 h post-infection. In wild type cells, NP staining was bright and primarily observed in the nuclei, where viral replication takes place. In contrast, NP staining was largely absent in the nuclei of WDR7, CCDC115, and TMEM199 KO cells and was instead concentrated in punctate structures near the peri-nuclear regions (Fig. 6b). The NP punctate structures co-stained partly with LAMP1, suggesting that at least a sub-fraction of the incoming virions are retained in the lysosomes. In contrast, NP staining was also reduced in CMTR1 KO cells but found in the nuclei like in wild type cells. Taken together, these suggested that incoming virions are blocked prior to nuclear entry and are likely retained in the endo-lysosomal compartments due to lack of viral fusion.

Although an acidic endo-lysosomal environment is required for viral fusion, studies have shown that exposure to pH lower than the optimal fusion pH may cause HA inactivation and coagulation of viral ribonucleoproteins (RNP)^53–55^. In addition, perturbation of V-type ATPase activity and localization can disrupt the pH gradient from early to late endosomes^56^ which IAV requires for efficient uncoating^57^. To test if the block in IAV infection is due to sub-optimal fusion pH, we compared the infectivity of two different H3N2 viral strains in the polyclonal KO cells. The X:31 strain has been shown to initiate membrane fusion at a lower pH and is more acid stable than the Udorn strain^53^. We thus hypothesized that X:31 virus will be less affected by the lower endo-lysosomal pH in WDR7, CCDC115, and TMEM199 polyclonal KO cells than Udorn virus. Consistent with this, we observed comparable viral NP RNA levels in KO and wild type cells at 16 h post-infection by X:31 virus. In contrast, KO cells have significantly lower viral RNA levels compared to wild type cells when infected with Udorn virus (Fig. 6c).

### CMTR1 is required for IAV cap snatching

**Fig. 7:**
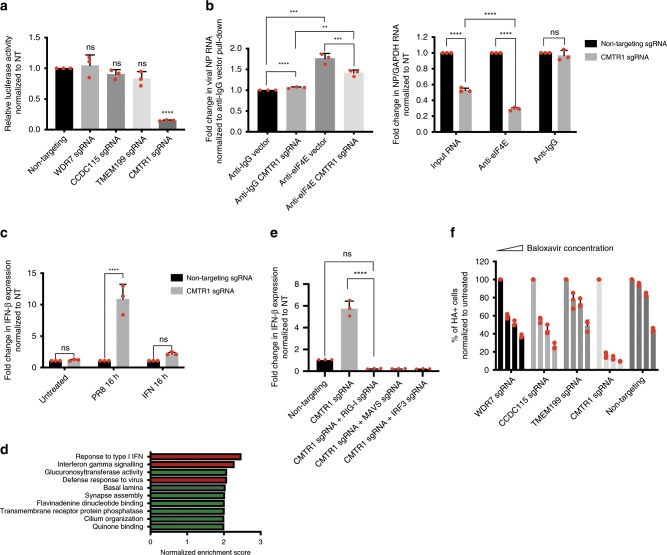
Loss of CMTR1 inhibits viral replication and up-regulates anti-viral genes. **a** A549 cells transduced with gene-specific or non-targeting sgRNAs were transfected with PR8 PA, PB1, PB2, and NP plasmids together with green-Renilla and Luciferase reporter plasmids for 24 hours. Luciferase activity was measured and normalized to A549 cells transduced with non-targeting sgRNA. Error bars represent standard deviations from three independent experimental replicates. **b** A549 cells transduced with CMTR1 or non-targeting sgRNA were infected with PR8 virus, in vivo crosslinked, lysed and immunoprecipitated with either anti-eIF4E or anti-IgG antibody to extract capped cellular and viral RNA. qRT-PCR was performed to monitor the relative abundance of PR8 NP RNA. (Left): Fold change in NP RNA levels normalized to anti-IgG non-targeting sgRNA pulldown. (Right): Fold change in NP relative to GAPDH RNA levels normalized to non-targeting sgRNA. Error bars represent standard deviations from three independent experimental replicates. **c** Fold change in IFN- β relative to GAPDH mRNA levels measured by qRT-PCR. A549 cells transduced with CMTR1 or non-targeting sgRNA were either mocked treated, infected with PR8 virus or treated with 200 U/ml IFN- β for 16 h. Fold change was normalized to A549 cells transduced with non-targeting sgRNA. Error bars represent standard deviations from three independent experimental replicates. **d** Gene Ontology (GO) enrichment analysis showing the top 10 up-regulated gene categories in A549 cells transduced with CMTR1 sgRNA versus non-targeting sgRNA after PR8 virus infection. IFN-related and antiviral gene categories are colored in red. **e** Fold change in IFN- β relative to GAPDH mRNA levels measured by qRT-PCR. A549 cells were transduced with CMTR1 sgRNA alone or together with sgRNA targeting RIG-I, MAV or IRF3 and infected with PR8 virus. Fold change was normalized to cells transduced with non-targeting sgRNA. Error bars represent standard deviations from three independent experimental replicates. **f** A549 cells transduced with gene-specific or non-targeting sgRNA were pre-treated with 0, 1, 5 or 10 nM of Baloxavir followed by PR8 virus infection. Y-axis shows the percentage of HA-positive cells normalized to untreated cells. Error bars represent standard deviations from three independent experimental replicates. ****P = 0.0001, **P < 0.01, by one-way ANOVA test.

CMTR1 was recently discovered as the human 2′-O-ribose cap methyltransferase^58,59^, which adds a methyl-group to the 5′-7 methylguanosine cap of eukaryotic mRNA to form the Cap1 structure (methylation of the 2′-O ribose of the first transcribed nucleotide). Since 2′-O-methylation of the mRNA cap has been known to be important for IAV cap snatching^60,61^, we hypothesized that loss of CMTR1 would inhibit viral transcription by preventing efficient cap snatching. To test this, we transfected WDR7, CCDC115, TMEM199 and CMTR1 polyclonal KO cells with a vRNA luciferase reporter construct carrying PR8 promoter and UTR regions, as well as plasmids expressing PR8 polymerase subunits PA, PB1 and PB2^62^. Twenty-four hours post-transfection, the cells were lysed and luciferase activity was measured. Consistent with our hypothesis, we observed lower luciferase activity in CMTR1 KO cells but not in WDR7, CCDC115, TMEM199 KO cells or wild-type cells (Fig. 7a).

To test the hypothesis that CMTR1 is involved in IAV cap snatching, we infected wild type and CMTR1 polyclonal KO cells with PR8 virus and immuno-precipitated the cell lysate with anti-eIF4E antibody to pull down capped viral and host RNA^63^. Relative abundance of pulled-down NP RNA in CMTR1 KO and wild type cells was then measured by qRT-PCR. Our results showed that while there was no difference in amount of NP RNA pulled down by anti-IgG control, there was significantly more NP RNA pulled down by anti-eIF4E antibody in wild type cells compared to CMTR1 KO cells, suggesting that the latter has less capped viral RNA (Fig. 7b). Since eIF4E binds to both cap0 and cap1 RNA, while IAV only efficiently cap snatch cap1 RNA, we normalized the amount of pulled-down NP RNA (cap1) against GAPDH (cap0 + cap1). We then compared this ratio between wild type and CMTR1 KO cells in anti-eIF4E and anti-IgG pulldown samples. We found that CMTR1 KO cells have a lower ratio of NP: GAPDH than wild type cells (Fold change = 0.29) in anti-eIF4E pulldown samples but not in anti-IgG control (Fig. 7b). Importantly, we found this difference to be more pronounced than that observed for un-precipitated input samples (fold change = 0.53), suggesting that the difference observed between CMTR1 KO cells and wild type cells is not just due to inhibition of viral replication. Together, these observations led us to conclude that CMTR1 is required for efficient IAV cap snatching.

### Loss of CMTR1 increases expression of anti-viral genes

Although Cap1 is present on most eukaryotic mRNAs, its precise functions are poorly understood as the lack of CMTR1 does not seem to have a significant impact on global protein translation^58^^.^ Recent studies have proposed that 2-O-ribose methylation of the mRNA cap acts as a mechanism by which the cell differentiates between self- and non-self RNA^64,65^, as siRNA knockdown of CMTR1 was shown to elevate Type I IFN response in A549 cells in the absence of additional stimulus. We hypothesized that the loss of CMTR1 may block IAV infection by both preventing efficient cap snatching and increasing cell autonomous antiviral responses. To test the latter, we measured the transcript levels of the anti-viral cytokine, IFN-β, in CMTR1 polyclonal KO cells and wild type cells in the presence and absence of PR8 infection via qRT-PCR. Interestingly, we observed an increase in IFN-β expression in CMTR1 KO cells but only when they were infected by PR8 virus (Fig. 7c), despite lower level of viral NP RNA detected in CMTR1 KO cells compared to wild type cells (Supplementary Fig. 10A). We also observed lower level of NS1 RNA in infected CMTR1 KO cells, which may help to explain the increase in IFN signatures in these cells. To confirm our results, we extracted RNA from CMTR1 KO cells and wild type cells with and without PR8 infection and performed bulk RNA sequencing. Principal component analysis (PCA) revealed significant differences in RNA expression profile between CMTR1 KO cells and wild type cells in the presence of PR8 infection but not at the resting state (Supplementary Fig. 10B). A closer inspection of the differentially-expressed genes showed an enrichment of Type I and II IFN-related genes as well as other antiviral genes in CMTR1 KO cells (Fig. 7d). To test if the increase in IFN signature is mediated by the RIG-I sensing pathway^66^, we transduced CMTR1 KO cells with sgRNA targeting RIG-I, MAV or IRF3 followed by infection with influenza PR8 virus. We found that the increase in IFN- β expression isield completely abrogated in the absence of RIG-I, MAV or IRF3, indicating that an intact RNA sensing pathway is required for the elevated IFN response in CMTR1 KO cells (Fig. 7e).

### Synergistic action between CTMR1 knockout and Xofluza

The recent FDA-approved drug Xofluza (Baloxavir Marboxil) blocks IAV infection by inhibiting the endonuclease activity of IAV PA subunit and preventing cap snatching^9,10^. To test if CMTR1 has potential interactions with Xofluza, we pre-treated WDR7, CCDC115, TMEM199, and CMTR1 polyclonal KO cells with increasing doses of Baloxavir (active form of the drug) prior to PR8 virus infection and measured changes in infectivity. While all four KO cell lines and wild type cells displayed a dose-dependent reduction in viral infection rate, CMTR1 KO cells demonstrated the most drastic decrease in infectivity with increasing dose of Baloxavir treatment (Fig. 7f). At the lowest concentration of drug administered (5 nM), CMTR1 KO cells had a 85% reduction in infectivity compared to 40%, 45%, 23% and 6% achieved in WDR7, CCDC115, TMEM199 KO cells and wild-type cells respectively. This indicated that loss of CMTR1 may confer synergistic protection against IAV infection with Xofluza treatment.

## Discussion

In this study, we identified 121 host genes that are required for IAV replication based on  our CRISPR screen. In addition, we devised and applied the MAIC algorithm to put these discoveries in the context of extensive previous literature on this topic, generating a ranked list of all known HDFs for influenza.

Unlike many earlier host factor screens that relied on cell survival as selection criterion, we adopted a different CRISPR/Cas9 screening strategy by using viral protein expression at an early time point post-infection as our phenotypic readout. Using such a continuous metric allowed us to identify a deeper set of HDFs (121 hits with FDR < 0.05) that play roles from early to late stages of the IAV life cycle. A significant fraction of our hits (77/121 hits) overlapped with those from previous RNAi screens and proteomics studies (Supplementary Data 3), including all six genes that were identified in at least four RNAi screens (ATP6AP1, ATP6V0C, ATP6V0D1, COPA, COPG, and NXF1)^25^. This differed from a previous published CRISPR/Cas9 cell survival screen which identified primarily early entry factors and shared few common hits with RNAi screens, suggesting that endpoint selection can strongly affect screening outcomes. Importantly, both our CRISPR/Cas9 screen and the recently published one^33^ identified shared HDFs absent from previous RNAi screens, indicating that our knowledge of IAV-host interactions had not yet reached saturation.

By deriving an information content weighting directly from the overlap between input data sets, the MAIC algorithm provided a systematic meta-analyses of multiple experiments and other data sources of unknown quality, aggregated both ranked and unranked data sources, and outperformed other methods in realistic comparisons using synthetic data, and in an experimental comparison using our CRISPR screen as a validation dataset. The ability to combine ranked and unranked data, and to systematically weight input data by a data-driven quality metric, overcomes some limitations of previous work^36,37^. Interestingly, our meta-analysis highlighted many relevant hits found in the *Drosophila* RNAi screen^16^ compared with other RNAi screens. In contrast, we found that there was relatively little relevant information content detected among a set of human genes under recent positive selection^67^. The MAIC approach revealed many HDFs supported by CRISPR or siRNA evidence, with strong evidence supporting a direct interaction with viral proteins, but with no existing annotation in the KEGG^35^ or FluMap^68^ databases. Strongly-supported examples include the *PRPF8* gene, which has recently been shown by another group to have a dose-dependent relationship with influenza virus expression^69^, as well as numerous genes, such as the splicing factor *SRSF6* and the elongation factor *EEF1A1* which have not, to our knowledge, been studied in influenza virus infection models. MAIC thus highlights genes that are strongly supported by evidence to play important roles in IAV infections, but have not been extensively studied previously.

We focused on genes highly ranked in our screen but not previously investigated in the context of IAV infection for functional follow-up experiments. Three of our top ranked hits from the CRISPR screens, *WDR7, CCDC115* and *TMEM199*, have been reported as putative V-type ATPase-associated co-factors^41,42,70,71^, but their functions in mammalian cells and especially in the context of viral infections are poorly understood. Here, we provide evidence that all three genes are required for efficient V-type ATPase assembly and IAV entry. Unlike V-type ATPase subunits, knocking out WDR7, CCDC115 or TMEM199 did not result in loss of cell viability, suggesting that these co-factors could serve as better therapeutic targets.

The unexpected observation that WDR7, CCDC115, and TMEM199 polyclonal KO cells underwent expansion and over-acidification of the endo-lysosomal compartments led us to hypothesize that long-term inhibition of V-type ATPases may cause a compensatory increase in lysosomal function, a phenomenon that is observed in cells that were subjected to starvation or prolonged treatment with lysosomoptropic compounds^47,72^. In support of this, we observed increased nuclear translocation of TFEB and expression of lysosomal genes in WDR7, CCDC115, and TMEM199 KO cells. A previous study reported that knockdown of RNASEK, another V-type ATPase-associated factor, also led to increased endo-lysosomal acidification that was mediated by the P-type ATPase ATP13A2^73^, suggesting that other ATPase proteins may over-compensate for the inactivation of specific V-type ATPases. TFEB over-expression has been postulated as potential treatment for a variety of human diseases^74–76^. Our observation that TFEB-mediated expansion and over-acidification of endo-lysosomal compartments block IAV and VSV infection opens the possibility of upregulating TFEB activity as a treatment option for reducing acid-dependent viral infections.

While it has been established that an acidic endosomal environment is required for IAV entry^51,77^, we showed that depletion of WDR7, CCDC115, and TMEM199 increases endo-lysosomal acidification, yet reduces viral infection. We hypothesized that this could be due to two reasons: First, it has been reported that exposure to pH that is too acidic could lead to HA inactivation and inhibition of viral fusion^53,55^. In support of this, we showed that incoming virions were trapped in punctate structures around the perinuclear regions of WDR7, CCDC115, and TMEM199 polyclonal KO cells, which partially co-stained with LAMP1. We also observed increased degradation of endocytic cargo in these cells, suggesting that incoming virions which failed to fuse could be targeted for degradation in the endo-lysosomes. In addition, we found that X:31 virus, a more acid stable H3N2 strain than the Udorn virus^53^, retained normal infection rates in WDR7, CCDC115 and TMEM199 polyclonal KO cells. Second, sequential exposure to lower pH from the early to late endosome has been shown to be required for productive IAV infection^45,57^. Depletion of WDR7, CCDC115, and TMEM199 may thus block IAV infection by disrupting the pH gradient in the endo-lysosome pathway^56^. Here, we showed that BafA treatment, while reducing lysotracker red staining, did not restore IAV infection in WDR7, CCDC115, and TMEM199 polyclonal KO cells. The lysotracker red signal in the KO cells also co-stained with both late endosome and lysosome markers, indicating possible homogenization of pH across different endosomal compartments.

IAV relies on a unique strategy of cap-snatching to carry out viral transcription and replication^78^. The PA subunit of the IAV polymerase complex functions as a cap-dependent endonuclease, which recognizes and cleaves short fragments of capped host mRNA to use as primer for its own mRNA synthesis^79^. Although it has been long appreciated that 2′O-ribose methylation of the host mRNA cap is required for efficient recognition and cleavage by PA^60,61^, no cap methyltransferase had been identified in IAV genetic screens to date. In this study, we discovered CMTR1 as an important IAV HDF, whose absence confers resistance against IAV infection by blocking viral cap snatching. We also observed that depletion of CMTR1 resulted in increased IFN response in IAV-infected cells. Unlike a previous study which showed that siRNA knockdown of CMTR1 causes up-regulation of IFN-β in the absence of additional stimulation^65^, we found differential expression of type I IFN and IFN-stimulated genes (ISGs) only when the cells were infected with IAV. We speculate that this is due to their use of siRNA, which could lead to siRNA-induced innate immune sensing by RIG-I/MDA5. The lack of immune activation in CMTR1 KO cells at resting state makes it a good drug candidate due to its therapeutic window and low risk of autoimmunity. Previous studies have shown that the IFIT family of antiviral proteins sequester 2′-O-unmethylated capped RNA and block viral protein translation^80,81^. Coincidentally, we observed an increase in IFIT gene expression in CMTR1 polyclonal KO cells, suggesting that inhibition of viral replication might be attributed to both cap snatching blockade and direct sequestration of viral RNA.

The advantage of targeting IAV cap snatching as a therapeutic strategy is best highlighted by the recent FDA approval of Xofluza (Baloxavir Marboxil), a small molecule drug that inhibits the endonuclease function of PA^9,10,55,82^. A single dose of Xofluza treatment has been shown to accelerate symptom alleviation and reduce viral load to a greater extent compared to the neuraminidase inhibitor Oseltamivir. Despite its effectiveness, resistant viral strains with reduced susceptibility to Xofluza have already been isolated in cell culture and clinical trials, raising concerns for long term administration of the drug^9,83^. Given that CMTR1 is required for efficient viral cap snatching, we tested for potential interaction between CMTR1 and Baloxavir. Our results provided preliminary evidence that depletion of CMTR1 confers synergistic protection again IAV infection with Baloxavir treatment. A combination therapy targeting both host CMTR1 and IAV endonuclease may thus serve as an attractive therapeutic option given greater barrier against drug resistance.

In conclusion, our study has identified and validated a number of HDFs that play important roles during IAV infection. We show that WDR7, CCDC115, and TMEM199 regulate V-type ATPase assembly and their absence causes compensatory expansion and over-acidification of the endo-lysosomal compartments, which hamper IAV entry. We also report CMTR1 as a novel HDF that is required for efficient viral cap snatching and regulation of cell autonomous immune response. Lastly, our MAIC algorithm consolidates data from all previous genetic screens and proteomics studies and generates an annotated list of IAV HDFs which can serve as useful resource for future studies.

## Methods

### Cell culture, reagents, and virus strains

A549, A549-Cas9, and 293T cells were cultured in Dulbecco’s Modified Eagle Medium (DMEM, Thermofisher) supplemented with 10% heat-inactivated fetal bovine serum (Sigma), 2 mM L-Glutamine (Gibco) and 1% penicillin. A549 and 293T cells were obtained from ATCC. A549-Cas9 cell line was generated by transducing A549 cells with a lentiviral construct (pXPR101) expressing Cas9 and Blasticidin deaminase. Cas9 activity was confirmed by transducing A549-Cas9 cells with a lentiviral construct (pXPR_011-sgEGFP) expressing eGFP and an sgRNA specific for eGFP. Polyclonal population of the A549-Cas9 cell line was used for the CRISPR screen to maintain heterogeneity of the cells. Primary NHLF cells were cultured in Mesenchymal Stem Cell Growth Medium (MSCGM, Lonza). PR8/A/34, A/Udorn/72 and A/Aichi/68 (X:31) Influenza A viruses were grown in MDCK cells in serum-free DMEM supplemented with 1% BSA and 1 μg/ml TPCK trypsin. GFP-Vesicular stomatits virus (VSV) was kindly.pngted by Dr. Sean Whelan’s lab. Influenza A/New Caledonia/20/1999, A/California/04/2009 and A/Vietnam/1203/2004-PR8-IBCDC-RG/GLP viruses were kindly.pngted by Dr. Daniel Lingwood’s lab. Bafilomycin A1 was obtained from invivogen (88899-55-2). Chloroquine diphosphate was obtained from Sigma (C6628). Baloxavir was obtained from MedChemExpress (HY-109025A).

### Plasmids

pXPR101 and pXPR_011-sgEGFP used to generate A549-Cas9 cells and pLentiGuide-puro (Addgene #52963) for secondary screen were provided by the Broad Institute Genetic Perturbation Platform. Individual sgRNAs were cloned into pLentiCRISPR-V2(Addgene #52961) and pXPR_004 (Puromycin resistance gene in pLentiCRISPR-V2 was replaced by eGFP) for validation in A549 cells and NHLFs respectively. For rescue experiments, the Cas9 gene in pXPR101 was replaced by codon-mutated versions of WDR7, CCDC115, TMEM199, and CMTR1 genes (pXPR101_rescue). For pseudovirus production, we used MLV Gag-pol, GFP, PR8 HA, PR8 NA, and MLV Env plasmids (kindly provided by Michael Farzan and Wayne Marasco).

### Antibodies

The following antibodies were used throughout this study: From EMD Millipore, Anti-Influenza A HA (AB1074) (1:200), FITC Anti-Influenza A Nucleoprotein clone A1 (MAB8257F) (1:200). From Abcam, Anti-LAMP1 clone H4A3 (ab25630) (1:100), Anti-Rab7 Alexa-Fluor647 clone EPR7589 (ab198337) (1:100), Anti-ATP6V0D1 (ab56441) (1:2000), β-actin antibody (ab6276) (1:10000). From BD bioscience, FITC mouse anti-human CD71 (555536) (1:100). From Thermofisher, Alexa-Fluor488 Goat anti-mouse IgG (1:500), Alexa-Fluor488 Donkey anti-goat IgG (1:500). From Sigma Aldrich, Anti-Flag M2 antibody (F3165) (1:2000). From Cell Signaling Technology, TFEB antibody (#4240S) (1:2000), Phospho-TFEB antibody (Ser211) (#37681S) (1:2000), Cox-IV antibody (4850s) (1:2000), HSP90 antibody (#4874) (1:2000) and TBP antibody (#8515) (1:2000). From Abnova, Anti-ATP6V1A (H00000523-A01) (1:2000).

### Pooled genome-wide CRISPR screen

Hundred million A549-Cas9 cells were transduced with the AVANA-4 lentiviral library^34^ to achieve 40% infection rate and average 500-fold coverage of the library after selection. After 24 h, the cells were selected with puromycin and an initial pool of 40 million cells were harvested for genomic DNA extraction using the Qiagen Blood and Tissue extraction kit according to manufacturer protocol. On day 9 post-transduction, 200–400 million puromycin resistant A459-Cas9 cells were infected with Influenza A PR8 virus at MOI5 for 16 h. They were then washed and stained with florescent anti-Influenza A HA (AB1074) antibody. HA-positive and HA-negative cells were sorted by FACS and harvested for genomic DNA. PCR of gDNA was performed in 100 μl reactions to attach sequencing adaptors and barcode samples. Each reaction consisted of 50 μL gDNA plus water, 40 μL PCR master mix and 10 μL of a uniquely barcoded P7 primer (stock at 5 μM concentration). Master mix comprised of 75 μL ExTaq DNA Polymerase (Clontech), 1000 μL of 10x Ex Taq buffer, 800 μL of dNTP provided with the enzyme, 50 μL of P5 stagger primer mix (stock at 100 μM concentration), and 2075 μL water. PCR cycling conditions: an initial 1 min at 95 °C; followed by 30 s at 94 °C, 30 s at 52.5 °C, 30 s at 72 °C, for 28 cycles; and a final 10 min extension at 72 °C. Samples were purified with Agencourt AMPure XP SPRI beads according to manufacturer’s instructions (Beckman Coulter, A63880) and sequenced on a HiSeq2000 (Illumina).

### sgRNA library cloning and lentiviral production

The AVANA-4 library (74,700 sgRNAs targeting 18,675 genes and 1000 non-targeting sgRNA) was provided by the Broad Institute Genetic Perturbation Platform. For the secondary screen, a plasmid library containing 18,870 sgRNAs targeting the top 1000 ranked genes from the primary screen and 787 genes from cross-validation analysis as well as 1000 non-targeting sgRNAs was synthesized as oligonucleotides (Broad Institute Biotechnology Lab). The sgRNAs were cloned by Gibson Assembly into the pLentiGuide-Puro vector. To produce lentivirus, 293T cells were plated in a 6-well dish at 0.5 × 10^6^ cells per well. Transfection was performed using TransIT-LT1 (Mirus) according to manufacturer’s protocol and virus was harvested 48 h post-transfection.

### Influenza A virus and VSV infection

A549 or NHLF cells were inoculated with 300l (6-well plate) or 2 ml (T75 flask) of influenza A virus at MOI 5 for 1 hour at 37° in serum-free DMEM. The cells were then washed and replaced with fresh serum-free DMEM supplemented with 1% BSA for 16 h. Infection was subsequently monitored by FACS or plaque assay.

For VSV infection, A549 cells were inoculated with 300 μl (6-well plate) of VSV virus at MOI 1 for 1 h at 37° in complete DMEM. The cells were then washed and replaced with fresh DMEM for 16 h. Infection was subsequently monitored by FACS.

### Screen analysis

Read counts corresponding to each guide RNA were normalised to reads per million and and log transformed. Quantile normalisation was performed in R. In order to control for the marked heteroscedasticity (Fig. 1b), local z-scores, for pools of values with different read counts, were calculated for sliding bins of varying size. For any comparison of two samples from which *n* read counts [*x*] and [*y*] are derived (for example, the flu-permissive and control FACS pools), the null hypothesis is *x*_*i*_ = *y*_*i*_, where *i* is the ranked position in the list of read counts. The read count bin was determined from the shortest distance between any point (*x*_*i*_, *y*_*i*_) and the line *y* = *x*. Lower (*l*) and upper (*u*) limits of *n* sliding bins of size *b* were defined such that each bin contains *b* values:

- Where $$i \,{<}\, 0.5 \times b$$, *l* = 0, *u* = *b*
- In the middle of the list, $l = i - 0.5 \times b$, $u = i + 0.5 \times b$
- Where $$\left( {n - i} \right) \,{<}\, 0.5 {\times} b$$, $$l = n - b$$, *u* = *n*

Z-scores were then calculated within each of these bins. *p* values were calculated from the sum of z-scores for sgRNAs targeting a particular gene compared to a density function modeled on an empirical distribution of possible combinations of sgRNA z-scores permuted at least 1e8 times by randomly rearranging z-scores for all sgRNAs in the screen. In order to minimize false negatives and maximize the discovery power of our screen, we did not require more than one sgRNA per gene to be significantly over-represented in the influenza virus-permissive FACS pool (permissive set). We report an additional “robust” set of hits in which the empirical *p*-value for a given gene, derived from the remaining sgRNAs after the sgRNA with the greatest effect is removed (remainder p), is less than 0.05. FDRs were calculated using the Benjamini-Hochberg method in scipy stats v1.1.0.

### Meta-analysis by information content (MAIC)

The MAIC algorithm seeks to combine the information in a heterogeneous group of data sources, in the form of lists of genes implicated in similar processes. It creates a data-driven information weighting for each source to prioritise relevant information, and allows the systematic integration of both ranked and unranked gene lists.

In a superset *A* of *m* input sets $\{ L_1,L_2,L_3,...L_m\}$, such as experimental data sources, each input set contains *n* named entities $\{ e_1,e_2,e_3,...e_n\}$, such as genes. Each input set belongs to a particular type of data source, which may have its own hidden biases. For example, siRNA affects some genes more than others, and some proteins have a tendency to be highly-connected in protein-protein interaction networks. Hence each input set is assigned to one of K categories, $\{ C_1,C_2,C_3,...C_K\}$. The algorithm begins with the assumption that a set of true positives, *T*, exists, and that, for any entity *e*, membership of several data sets *L* belonging to independent categories *C* increases the probability that *e* is a member of *T*. Each one of the data sets $L_j,j = 1,...,m$, has three attributes:

1. set of *n*_*j*_ entities $L_j = \{ e_{j1},e_{j2},e_{j3},...\,e_{jn_j}\}$
2. a category, $$c_j \in \{ C_1,C_2,C_3,...C_K\}$$
3. a structure, $r_j \in \{ R,F\}$ where *R* is ranked and *F* is flat, or not ranked.

A score value for each one of the genes in *A* which is based on the “popularity” of the genes in the input datasets. A gene will get a higher score for being represented in many different categories, as compared to being represented in many different datasets at the same category.

Each input set *L* is assigned a weighting score *w* to quantify the evidence in *e* that derives from membership of. The weighting score *w* is itself defined as the sum of the scores assigned to each entity *e* within *L*. The starting value of *s* for any *e* is arbitrary - any numerical value can be chosen, without altering the final scores. For simplicity, the initial *s* for each *e* is set to 1. In order to prevent any single category (*C*) of data from biasing the results, each entity draws only one score (the highest score) from each category. If there is no score for this *e* in a particular *C*, the score assigned will be zero. In each iteration, the score of an entity i in a given category k is updated:

$$\begin{array}{*{20}{c}} {s_{ik} = {\mathrm{max}}\left\{ {w_j^L\left| \,{g_i} \right. \in L_j \wedge c_j = C_k} \right\}i = 1,...,n;k = 1,...,K} \end{array}$$

The score of an entity for this iteration is the sum of the scores in each one of the categories.

$$\begin{array}{*{20}{c}} {s_i = \mathop {\sum }\limits_{k = 1}^K s_{ik},i = 1,...,n} \end{array}$$

The weighting score given to a dataset is the square root of the average score of the genes belongs to this dataset.

$$\begin{array}{*{20}{c}} {w_j^L = \sqrt {\frac{{{\sum} {\left( {g_i \in L_j} \right)s_i} }}{{n_j}}} } \end{array}$$

These equations are iterated until the values for *w* for all input sets *L* are no longer changing (ie. each value for $w_j^L$ is changed within 0.01 compared with the previous value.)

Some input data sources provide gene lists that are ranked according to the strength or statistical significance of experimental results. With descending rank, the probability that a given gene is a true positive result is expected to decrease. This decline in information content is modeled by fitting exponential decay curve to the measured information content at each position in a ranked list. The information content is inferred from the MAIC algorithm by truncating the list at every position and calculating a weighting ($w_j^L$) for the list up to that point, as if it were unranked. A specific weighting for each position in a ranked list is then calculated from the exponential decay function specific to this list.

Code to run the MAIC algorithm, and an online service with a user interface is present at https://baillielab.net/maic.

### Evaluation of MAIC

In order to evaluate MAIC against existing methods, we built a simulated data generator to generate ranked, unranked, and mixed data based on the Thurstonian ranking model^84^ which ranks entities by figure Z in descending order for each entity generated from Gaussian distribution with mean value μ and variance of the square of σ. Then we cut the generated lists by leaving only 0.5% (2) entities and labeled the list as ranked or unranked. The total ratio for real entities among all entities is also 10% in this evaluation. In the case of the present work, an entity is a protein-coding gene in the human genome. We use the term “entity” here because the approach is generalizable to a broad range of applications.

For List_*i* (*i* = 1…*n*), Entity_*k* (*k* =1…*m*), mean_noise *M*, the score *Z* for Entity_*k* is:

$${Z}_{k}\sim {N}\left( {{\mu}_{k},{\sigma}_{i}^2} \right)$$

$${\mathrm{Log}}\left( {\sigma _i} \right) = {\mathrm{log}}\left( {M} \right) + {t}$$

$${t}\sim {N}\left( {0,{h}^2} \right)$$

$${D} = {h}^2$$

We used MAIC, robust tank aggregation (RRA)^37^, and a simple vote counting (VC) method ranking entities by frequency on this model. We used top-25 overlap ratio (classification accuracy) as the metric of success, comparing the top-25 entities of result with top-25 true entities ranked by *μ*_*k*_.

In evaluation experiments we tested MAIC against RRA and VC over the following variations in synthetic input data:

1. Noise: setting mean noise M (defining mean quality of lists instead of mean numerical value of σ) among input lists. M was varied in the range [0,0.1,0.5,1,3,12], from perfect data without noise to data with very high noise.
2. Heterogeneity: setting D [0 to 3] to show variability of the quality of input lists, in order to model the real-life scenario in which data quality and relevance from different experiments is expected to vary widely. D is varied from 0 (indicating the same noise level (data quality) for all lists) to  3 (indicating very heterogeneous noise levels among the input lists). Importantly, heterogeneity is varied independently of the average noise among all input lists.
3. Ranked:unranked ratio: setting the ratio of the number of ranked lists to be 50% and 100%, leaving the remaining lists as unranked.

### Gene set enrichment analysis (MAIC output)

Gene set enrichment analysis was performed on gene MAIC score ranks, using package ‘fgsea’ in R version 3.5.2. 10^6^ permutations were used to derive *p*-values, and the Benjamini-Hochberg method was used to control false discovery rate (<0.05). The following gene set libraries were queried: KEGG 2016, BioCarta 2016, Reactome 2016, WikiPathways 2016, NCI Nature 2016, GO Biological Process 2018, GO Molecular Function 2018 and GO Cellular Component 2018. Reference for FGSEA (note no PMCID yet as only on bioRxiv): Sergushichev A (2016). “An algorithm for fast preranked gene set enrichment analysis using cumulative statistic calculation.” bioRxiv. 10.1101/060012, http://biorxiv.org/content/early/2016/06/20/060012.

### Validation of individual hits using gene-specific CRISPR sgRNA

For validation of individual hits in A549 cells, the two best sgRNAs from the AVANA-4 library were cloned into pLentiCRISPR-V2 and lentivirus was produced from 239T cells as described above. A549 Cells were transduced and selected with 1 μg/μl Puromycin for 8 days and genome-editing was confirmed by deep sequencing and CRISPResso analysis. For validation in NHLF cells, sgRNAs were cloned into pXPR_004, which carries eGFP instead of Puromycin resistance gene. Following transduction, GFP+ NHLF cells were sorted by FACS. GFP+ cells were infected with influenza A virus for 16 h at MOI5 and stained for surface HA using anti-influenza A HA antibody (AB1074).

### Rescue and over-expression of the KO genes

For the rescue experiment, A549 cells were transduced with pLentiCRISPR-V2 expressing a gene-specific sgRNA together with a XPR101_rescue plasmid expressing Flag-tagged codon-mutated version of the gene. Cells were selected with 1 μg/μl Puromycin and 10ug/ul Blasticidin for 8 days. Expression of the add-back gene was confirmed by Western Blot. To test if the genes of interest have redundant functions, A549 cells were transduced with different combinations of the gene-specific sgRNAs and codon-mutated versions of the genes. To test the effect of over-expressing the genes alone, A549 cells were transduced with the rescue plasmids in the absence of sgRNAs.

### Flow cytometry

Cells were stained with antibodies in PBS + 1% BSA for 30 min on ice and fixed with 4% paraformaldehyde. For intracellular staining for Influenza A nucleoprotein (NP), cells were fixed and permeabilized using 0.1% Saponin (Sigma Aldrich) prior to antibody staining. Data were acquired on the BD Accuri (Bd Bioscience) and analyzed by FlowJo software (TreeStar).

### Western blotting

To check for expression of WDR7, CCDC115, TMEM199, and CMTR1 expression in rescue experiments, 5 × 10^5^ transduced cells were washed with ice-cold PBS and lysed in RIPA buffer (Thermofisher) supplemented with EDTA-free Protease inhibitor cocktail (Roche). Cell lysates were span at 12,000 rpm in a microcentrifuge for 10 min at 4 °C and denatured by heating at 95 °C in SDS loading buffer + DTT. Proteins were separated on a NuPAGE Novex 12% Tris-Glycine gel and transferred to a polyvinylidene difluoride membrane (Milipore). Immunoblotting was performed according to standard protocols using Rabbit Anti-Flag primary antibody and HRP-conjugated anti-rabbit secondary antibody.

To check for TFEB and Phospho-TFEB expression, cytoplasmic and nuclear proteins were extracted from 5 × 10^5^ cells using the NE-PER Nuclear and Cytoplasmic Extraction reagents (Thermo Scientific) according to manufacturer protocol. Immunoblotting was performed according to standard protocols using anti-TFEB and anti-phospho-TFEB primary antibodies and HRP-conjugated anti-rabbit secondary antibody.

### RNA-extraction and qPCR

Total RNA was extracted from 1 × 10^5^ cells using the RNeasy Mini Kit (Qiagen) according to manufacturer’s protocol. First strand cDNA synthesis was performed using 500 ng of total RNA with the Superscript III First-strand Synthesis system with Oligo(dT) (Thermofisher). Quantitative qPCR was performed using the Q5 hot start high fidelity polymerase and SYBR green I Nucleic Acid Gel stain (Thermofisher) on the Roche 480 Light Cycler (Roche). Human GAPDH was used as reference normalization control and expression levels were quantified by the delta Ct method. Primer sequences are as follow:

Human IFN- β

F: 5′ – TGCTCTCCTGTTGTGCTTCT-3′

R:5′ – ATAGATGGTCAATGCGGCGT-3′

Influenza PR8 NP

F: 5′ – ATCGGAACTTCTGGAGGGGT-3′

R:5′ – CAGGACTTGTGAGCAACCGA-3′

Influenza PR8 NS1

F: 5′ – GTCTGGACATCGAGACAGCC-3′

R:5′ – GAGTCTCCAGCCGGTCAAAA-3′

Influenza A/New Caledonia/1999 HA

F: 5′ – TCACCCGCCTAACATAGGGA-3′

R:5′ – TGCAAAAGCATACCATGGCG-3′

Influenza A/California/2009 HA

F: 5′ – GGACACTAGTAGAGCCGGGA-3′

R:5′ – CAATCCTGTGGCCAGTCTCA-3′

Influenza A/Vietnam/2005 HA (H5N1)

F: 5′ – TGAGCGCAGCATGTTCCTAT-3′

R:5′ – GCCCGTTCACTTTGGGTCTA-3′

Human GAPDH

F: 5′ – GGGAGCCAAAAGGGTCATCA-3′

R:5′ – AGTGATGGCATGGACTGTGG-3′

### RNA sequencing

Transcriptomic analysis was performed using the Smart-Seq2 protocol. Total RNA was extracted using the RNeasy Mini Kit (Qiagen). cDNA was synthesized from 1 ng of total RNA using the SuperScript III reverse transcription system, followed by PCR pre-amplification and quality check using high-sensitivity DNA Bioanalyzer chip (Agilent). 0.15 ng of pre-amplified cDNA was then used for the tagmentation reaction carried out with the Nextera XT DNA sample preparation kit (Illumina) and final PCR amplification. Amplified library was sequenced on a Nextseq 500 (Illumina). For data analysis, short sequencing reads were aligned using Bowtie and used as input in RSEM to quantify gene expression levels for all UCSC hg19 genes. Data were normalized and analyzed using the R software package DESeq2.

### Plaque assays

A549 or NHLFs cells were infected with Influenza A PR8 or Udorn virus at MOI 0.1 in serum-free DMEM supplemented with 1% BSA and 1 μg/μl TPCK trypsin. 48 h post-infection, supernatant was collected and serial-diluted. Two hundred microliters of the diluted supernatant was used to infect MDCK cells on 6-well plates and the number of plaques were counted after 72 h. The virus titer was calculated in Plaque forming units (PFU)/ml.

### Proliferation assays

A549 cells were transduced with pLentiCRISPR-V2 expressing sgRNA against genes of interest and selected with 1 μg/μl Puromycin for 2 days. On day 3, 5000 puromycin resistant cells were re-seeded on 6-well plates and changes in total cell number were monitored on day 5, 7, and 9. On day 9, some cells were harvested for ALAMAR Blue assay (Thermofisher, DAL1025) and Annexin V staining (Thermofisher, V13241) according to manufacturer protocol.

### MLV-GFP pseudovirus production and entry assay

MLV-GFP pseudovirus was produced by transfecting 1 μg of MLV Gag-pol plasmid, 1 μg of GFP plasmids, 0.3ug of Influenza PR8 HA plasmid, 1.2 μg of NA plasmid or 1.2 μg of MLV-Env plasmid into 293T cells seeded on 6-well plate at 0.5 × 10^6^ cells per well. Virus was harvested and filtered 48 h post-transfection. To test for entry, A549 cells were spinoculated with the pseudovirus at 2000 rpm for 30 min. Before cell transduction, pseudovirus was incubated with 1ug/ml TPCK-treated trypsin for 1 h at room temperature and then mixed with trypsin-neutralizing solution. GFP expression was monitored 48 h post-spinoculation by FACS.

### Influenza A virus binding assay

Cells were seeded on 6-well-plates and inoculated with Influenza A PR8 virus at MOI 100 for 30 minutes at 4 °C. Cells were then washed twice with ice cold PBS and stained for surface HA using anti-influenza A HA antibody (AB1074).

### Measuring level of cell surface sialic acid

Cells were stained with Sambucus Nigra lectin (SNA) (Vector Laboratories Inc.) according to manufacturer protocol. Briefly, Cells were incubated with 10ug/ml FITC-conjugated Lectin at room temperature for 30 min. They were then washed twice in PBS and analyzed by FACS.

### Fluorescent-in situ-hybridization (FISH)

1 × 10^5^ cells were seeded on a chambered cover glass (VWR, Nunc Lab-Tec 2 wells) pre-treated with 0.1 mg/ml poly-D-lysine. The cells were infected with Influenza A PR8 virus the following day for 4 h at 37°. and then fixed and stained with Stellaris Quasar 570 RNA FISH probes against Influenza A PR8 NP RNA according to manufacturer protocol (LGC Biosearch Technologies). Images were taken on the Olympus FV1200 IX83 confocal microscope and percentage of RNA+ cells relative to the total number of cells was quantified.

### Confocal microscopy

Cells were imaged on the Olympus FV1200 IX83 laser scanning confocal microscope equipped with a 40X objective and LD559, LD635 and LD405 (Olympus Life Science). Images were taken using the Olympus FV software and analyzed using ImageJ. For imaging of lysotracker red, Lysosensor blue, Oregon Green Dextran, Rab7 and LAMP1, 1 × 10^5^ A549/NHLF cells were seeded onto chambered cover glass (VWR, Nunc Lab-Tec 4 wells) pre-treated with 0.1 mg/ml poly-D-lysine the day before. They were treated with 100 nM lysotracker dye for 1 h at 37 °C, followed by fixation with 4% paraformaldehyde and permeabilization with 0.1% Saponin. The cells were blocked with PBS with 1% BSA and 0.1% Tween20 for 1 h at room temperature and stained with anti-Rab7 and anti-LAMP1 antibodies overnight at 4 °C. The cells were then stained with secondary Alexa-fluor488-conjugated goat anti-mouse IgG antibody and DAPI for 1 h at room temperature. Images were acquired with a ×40 objective using the setup described above.

For visualization of Influenza NP localization within the cells, A549 cells were infected with Influenza A PR8 virus at MOI 200 for 2 h at 37°. Infected cells were then fixed with 4% paraformaldehyde and stained with FITC anti-influenza A NP antibody overnight at 4°. The next day cells were washed, stained with DAPI for 1 h at room temperature and images were acquired as described above.

### Measuring lysosomal degradation of DQ-BSA

1 × 10^5^ A549 cells were seeded on 12-well plates and incubated with 20ug/ml DQ Green BSA (Thermofisher, D12050), 100 nM Lysotracker Red and DAPI for 1 h at 37 °C. The cells were then washed in PBS and fixed in 4% paraformaldehyde. Confocal microscopy Images were acquired with a ×40 objective using the setup described earlier.

### Luciferase reporter assay for influenza A virus replication

To measure viral polymerase activity, we utilized a vRNA-luciferase reporter system. Briefly, A549 cells were transfected with a vRNA reporter plasmid expressing firefly luciferase under a viral UTR. The cells were also transfected with influenza A virus PA, PB1, PB2, NP, and Renilla. Twenty-four hours post-transfection, cells were lysed and mixed with Dual Glo substrate (Promega) according to Manufacturer’s protocol. Luminescence was measured and quantified using a Synergy H1 multi-mode microplate reader (BioTek).

### In vivo cross-linking coupled immunoprecipitation with anti-eIF4E antibody

Cells were harvested and cross-linked with 0.3% formaldehyde in culture media for 10 min at 37 °C to enable high stringency washes of the in vivo protein-RNA complexes^63,85^. Cells were washed three times with PBS and then fractionated into nuclear and cytoplasmic fractions. Extracts from the two fractions were combined and treated with Turbo DNase I (Ambion) and RNase inhibitor (NEB) prior to pre-clearing using protein-G agarose to remove non-specific contaminants that bind agarose. Anti-eIF4E (Cell Signaling) was used for immunoprecipitation. The immunoprecipitates were subject to heat inactivation at 56 °C for 15 min before subjecting to RNA isolation with 3 volumes of Trizol (Invitrogen).

### Reporting summary

Further information on research design is available in the Nature Research Reporting Summary linked to this article.

## Supplementary information

Supplementary Information

Reporting Summary

Description of Additional Supplementary Files

Supplementary Data 1

Supplementary Data 2

Supplementary Data 3

## Data Availability

Source data underlying Figs. 3A–F, 4A–B, 5C–5F, 6A and C, 7A–F, and Supplementary Figs. 5A–E, 6A and C, 7A–B, 8A–8C, and 10C are provided as a Source Data file. RNAseq data for Fig.7d, Supplementary Fig. 9B and Supplementary Fig. 10B have been uploaded on NCBI Gene Expression Omnibus (GEO) database (accession number: GSE141171). Interactive results from MAIC can be viewed at http://baillielab.net/maic/flu

## Source data

Source Data

## Code availability

A repository of the software developed for this project can be downloaded at https://github.com/baillielab/maic
