# Supplementary material for: Genome-wide CRISPR screen identifies host dependency factors for influenza A virus infection
Source: Nat Commun. 2020 Jan 9;11:164. doi: 10.1038/s41467-019-13965-x (PMC6952391; doi:10.1038/s41467-019-13965-x)
Supplement: Supplementary file 1 — Supplementary Information [file 41467_2019_13965_MOESM1_ESM.pdf]

Supplementary Information

Li *et al*

Supplementary Notes

Supplementary note 1 :

We performed a systematic comparison of statistical methods for CRISPR screen analysis. Our approach of combining empirical p-values ($\sum -log_{10}(p)$) sums the evidence in support of over-representation of sgRNAs targeting a given gene. Of the four sgRNAs targeting any gene, over-representation of any single sgRNA could potentially lead to a significant p-value under this method. This optimises the discovery power of the screen in the context of non-functional sgRNAs. We compared this approach to two other methods, STARS and MAGeCK. Our results show that our approach has similar discovery power to STARS compared to two gold-standard sources (the meta-analysis of siRNA screens by Tripathi *et al*[1], and MAIC meta-analysis of all data sources, excluding our CRISPR screen), but is more reproducible between two independent screens (Supplementary Figure 1).

Outputs were evaluated using the average overlap score (aos) - a variant of the sequence alignment quality measure developed here[29]. We chose this measure over other standard approaches (such as area under a receiver-operator curve) because it incorporates the absolute number of overlaps between two lists, as well as the respective rankings of each list. The AOS method calculates the overlap between two ranked lists iteratively, moving through each rank in the list and calculating the proportion of elements that overlap. The AOS is the average of all proportions in the lists.

Category	Datasource	Type	Ref
CRISPR_survival	Han_RD2	Ranked	[3]
CRISPR_survival	Han_RD5	Ranked	[3]
RNAi	Tripathi_Z_RSA	Ranked	[1]
RNAi	S4_host_Z_Konig	Ranked	[1,4]
RNAi	S4_host_Z_Karlas	Ranked	[1,5]
RNAi	S4_host_Z_Brass	Ranked	[1,6]
RNAi	S4_host_Z_Ward	Ranked	[1,7]
RNAi_confirmed	S4_host_SuHit	Ranked	[8]
RNAi_confirmed	S4_host_TranHit	Ranked	[9]
RNAi_confirmed	S4_host_ShapiraHit	Ranked	[10]
RNAi_confirmed	Kawaoka_knockdown_restricts_top	Ranked	[11]
RNAi_confirmed	Kawaoka_knockdown_restricts	Ranked	[11]
RNAi_drosophila	Hao_DOWN	Ranked	[12]
shRNA	Hacohen_lentivirus_shRNA	Ranked	
Annotated	KEGG(174)	Not ranked	[13]
Annotated	FluMap_Kawaoka	Not ranked	[14]
HaploidScreen	Carette	Not ranked	[15]
Positive_selection	SabetiOver125	Ranked	[16]
Genetic	CiancelliHorby	Not ranked	[17,18]
protein_interaction	S4_host_Viral_ShapiraY2H	Not ranked	[10]
protein_interaction	deChassey_y2h	Not ranked	[19]
APMS	S4_host_Viral_APMS	Not ranked	[1]
COIP	WatanabeCOIP	Not ranked	[11]
COIP	S4_host_COIP_Tripathi	Not ranked	[1]
incorporated	Hutchinson_incorporated	Not ranked	[20]
protein_interaction	Song_et_al	Ranked	[21]
protein_interaction	Shaw_et_al	Not ranked	[22]
protein_interaction	Tafforeau	Ranked	[23]
protein_interaction	Bradel-Trethaway	Ranked	[24]
protein_interaction	Heaton	Ranked	[25]
protein_interaction	Jorba	Ranked	[26]
protein_interaction	Lin	Ranked	[27]
Proteomics	Dove_Increased2fold	Ranked	[28]
Proteomics	Dove_Decreased2fold	Ranked	[28]
expression	Shapira_expression(1028)	Not ranked	[10]
coexpresssion	FANTOM5 coexpression clusters associated with influenza	Not ranked	[2]

Supplementary **note 2**: Sources of data used for MAIC. Where possible, data were obtained from the comprehensive meta-analysis by Tripathi *et al*[1]. Genome-wide screens were limited to a maximum of 2000 genes. Lists of "hits" identified by the authors of some screens using a variety of combinatorial statistical methods, were included as unranked lists, as in Tripathi *et al*[1]. FANTOM5 coexpression clusters (C270, C413, C3829, C3538, C0, C527, C1236, C1162, C4341, C2, C13, C1932, C6, C26, C447, C7, C187, C884, C2568, C3953, C2409) were included if they were associated with influenza in the original FANTOM5 paper.[2] Each gene derives only one MAIC score from a given data source category (See Methods, main paper).

Supplementary Figures

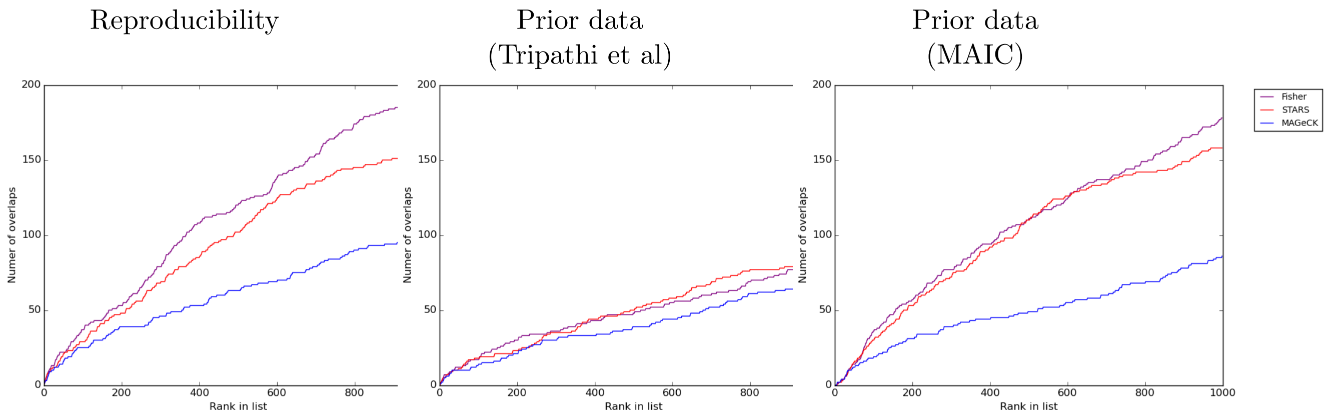

Supplementary Figure 1: CRISPR screen analysis by three different statistical methods. In each case the overlap between the CRISPR results and the top 1000 genes in another ranked list of genes is plotted on the y-axis for a given position in the results list (x-axis). This is analagous to a receiver-operator characteristic curve, but allows for a difference in the total number of overlapping genes between each list. Left panel (reproducibility) shows overlap between screen 2 and screen 1. Middle panel (Tripathi et al) shows overlap between the results of our combined CRISPR screens and a recent composite analysis of siRNA screens. Right panel (MAIC) shows overlap between combined CRISPR results and the results of a MAIC analysis from which the CRISPR results were excluded.

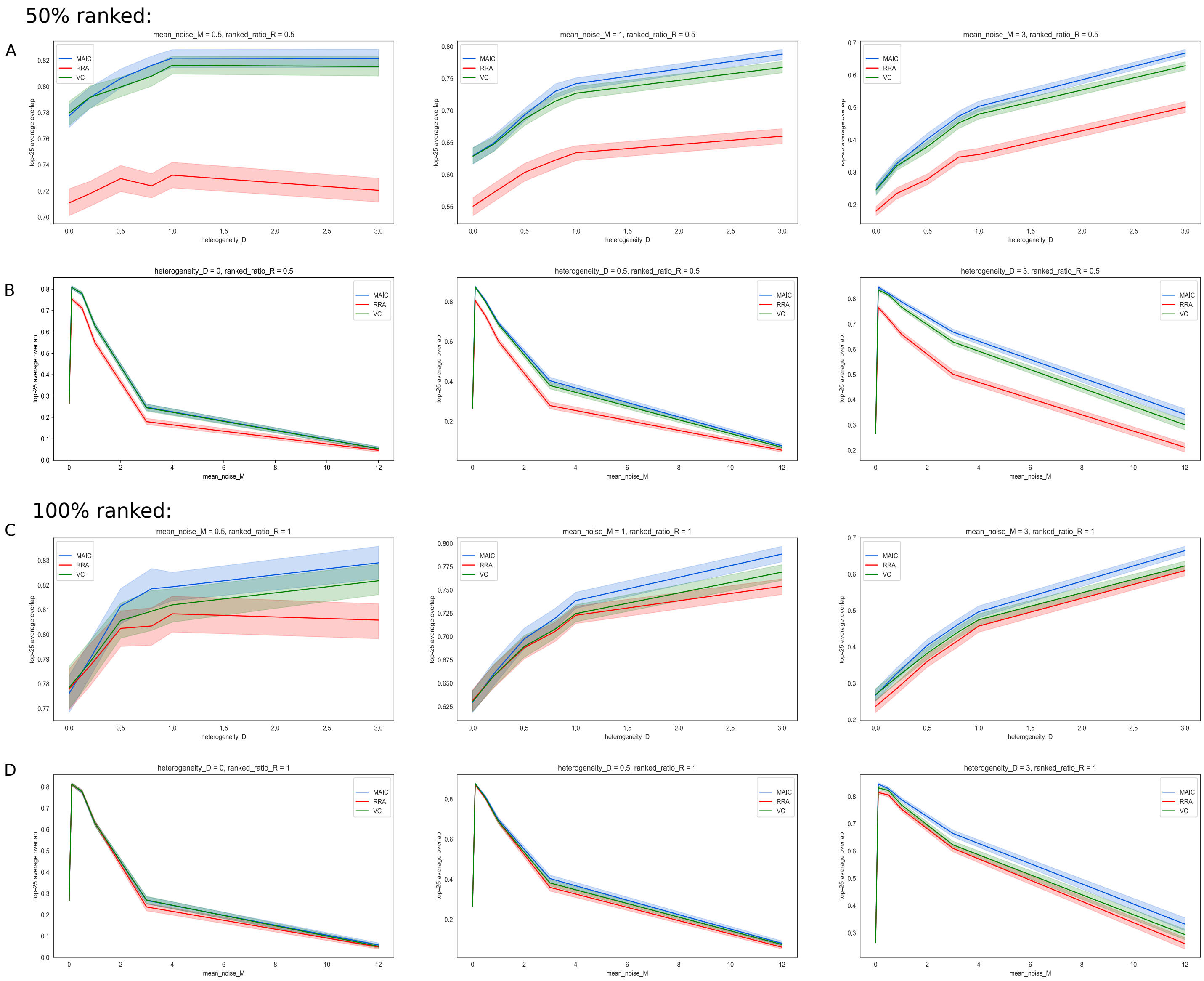

Supplementary Figure 2: *in silico* Evaluation of MAIC performance using synthetic data. y-axes show overlap ratio compared to known truth. MAIC (blue) is compared with existing methods (robust rank aggregation, RRA, red; and vote counting, VC, green; narrow line: mean of 100 replicates; shading: 95% CI). Results for a range of realistic inputs are shown: a mixture of ranked and unranked data sources (50% ranked, top two rows), or all ranked data sources (100% ranked, lower two rows). Rows (A,C) effect of change in heterogeneity (x-axis) for low, moderate and high levels of mean noise; (B,D) effect of change in noise (x-axis) for low, moderate and high levels of heterogeneity.

Final list of hits	%inhibition
ATP6V1A_2	58.1
COG4	54.5
CMTR1	51.4
WDR7	50.9
SLC35A1	48.6
COG3	45.4
CCDC115	41.8
DBR1	35.1
TMEM199	34.7
RABGGTB	34.4
GGPS1	29.9
PCID2	29.6
TRAPPC3	29.0
DPAGT1	26.1
SHFM1	25.5
PREB	23.0
NUDT21	22.7
YKT6	20.3
RABGGTA	15.2
TRAPPC8	15.1
TPR	14.1
SCAP	12.0
BCL2L1	11.7
MCM3AP	11.0
RPN2	11.0
ALG2	10.4
SRRT	8.8
SAP30BP	7.8
SPCS3	4.6
TNFAIP3	2.3

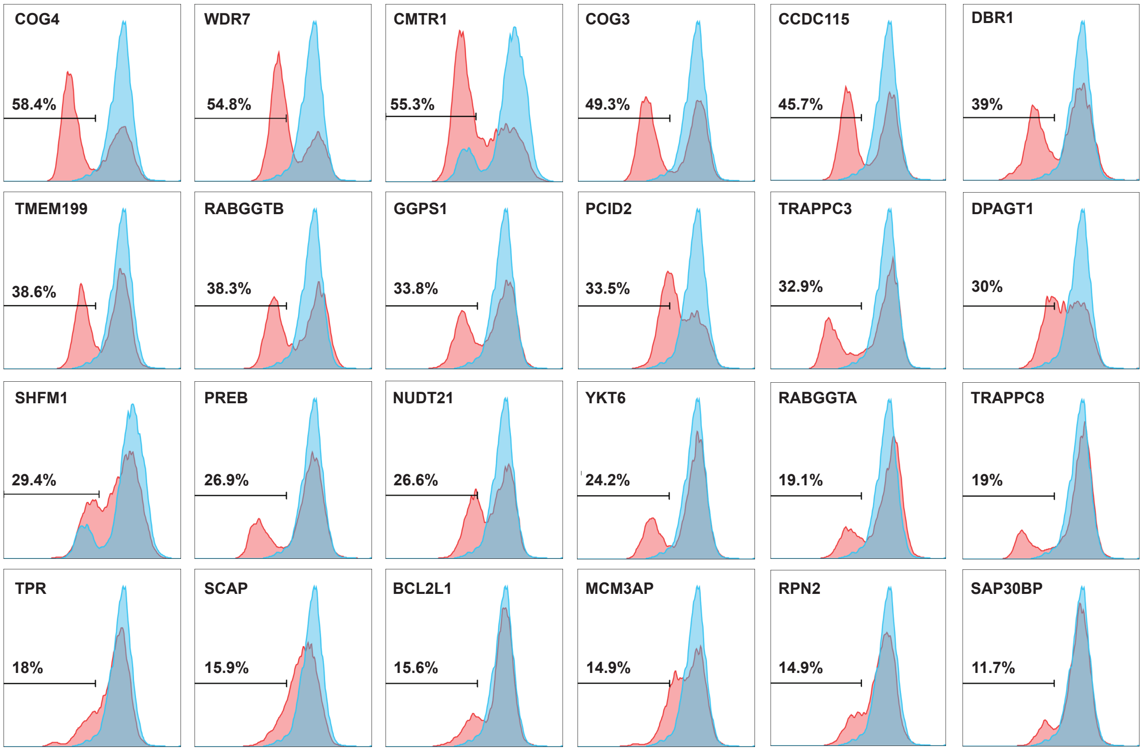

Supplementary Figure 3: Validation of screen hits in A549 cells using individual sgRNAs. Flow cytometry of surface HA expression in PR8 infected A549 cells transduced with either gene-specific (red) or non-targeting sgRNA (blue). Number shows percentage of HA-negative cells that were transduced with gene-specific sgRNAs. Histograms shown are representative of three independent experimental replicates.

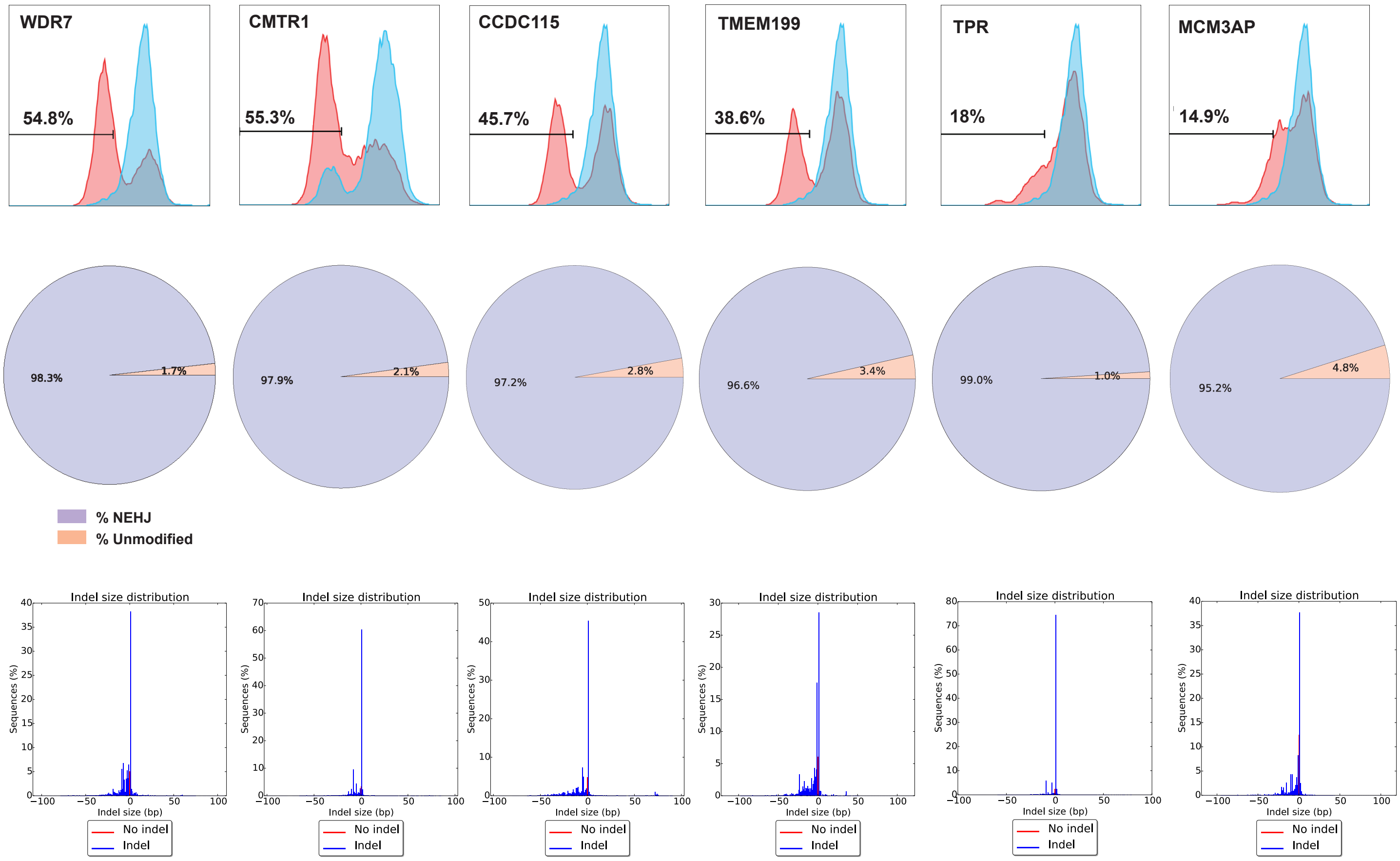

Supplementary Figure 4: Measuring genome editing efficiency. Genomic editing efficiency of selected CRISPR sgRNAs measured by massively parallel sequencing of the CRISPR target site and CRISPResso analysis. All transduced cells display high frequency of INDELs (>95%) despite different inhibitory effect on IAV infection.

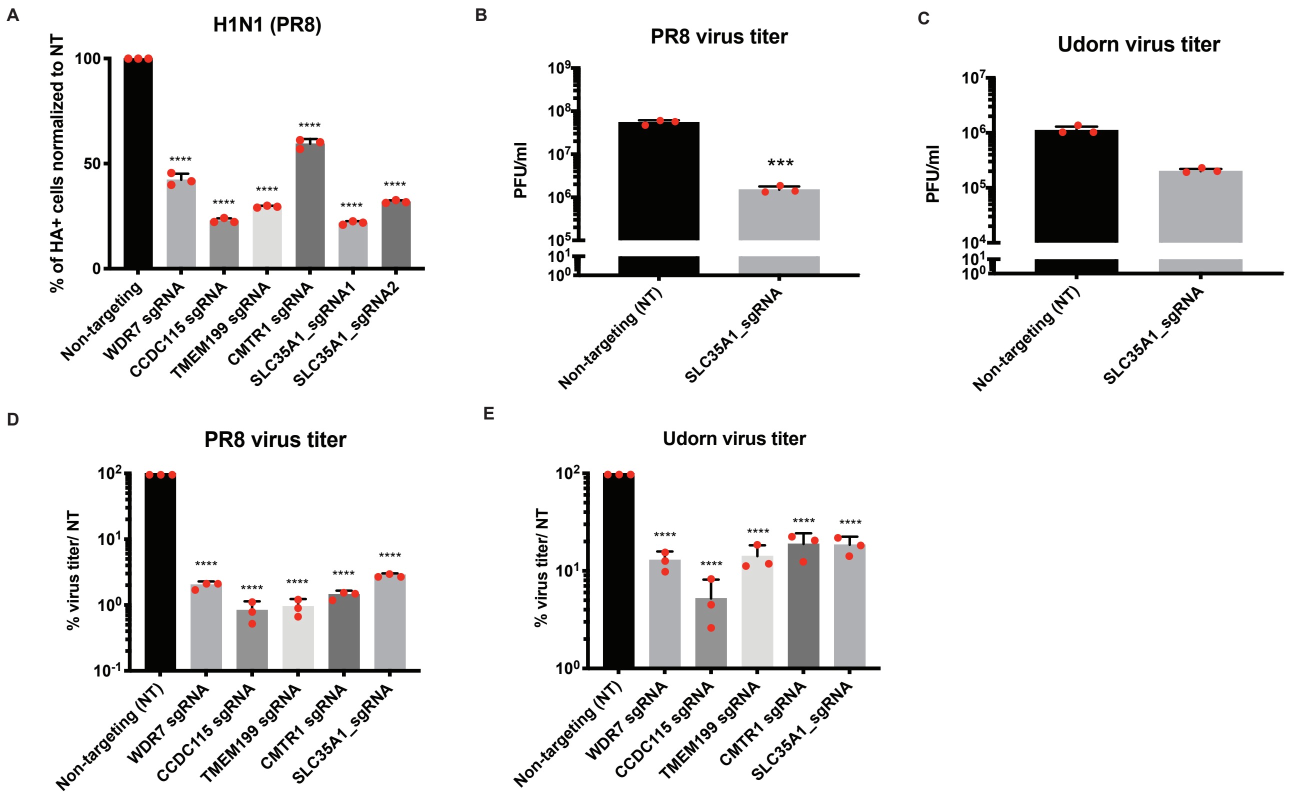

Supplementary Figure 5: Comparison of identified HDFs to SLC35A1. (A) A549 cells were transduced with either gene-specific or non-targeting sgRNA, followed by infection with PR8 virus at MOI 5 for 16 hours. Two different sgRNAs were used for SLC35A1. Y-axis shows percentage of HA-positive cells normalized to non-targeting sgRNA. Error bars represent standard deviations from three independent experimental replicates. (B) Virus titer in plaque forming units (PFU)/ml at 48 hours post-infection with PR8 or (C) Udorn virus at MOI 0.1. Supernatant used for the plaque assay was collected from infected A549 cells transduced with either non-targeting sgRNA or sgRNA targeting SLC35A1 (SLC35A1_sgRNA1 shown in Supp Fig.3B was selected for this experiment). Error bars represent standard deviations from three independent experimental replicates. (D) Virus titer in plaque forming units (PFU)/ml at 48 hours post-infection with PR8 or (E) Udorn virus at MOI 0.1. Supernatant used for the plaque assay was collected from infected A549 cells transduced with either gene-specific or non-targeting sgRNAs. Error bars represent standard deviations from three independent experimental replicates. ****P=0.0001, ***P<0.001 , **P<0.01, by one-way ANOVA test.

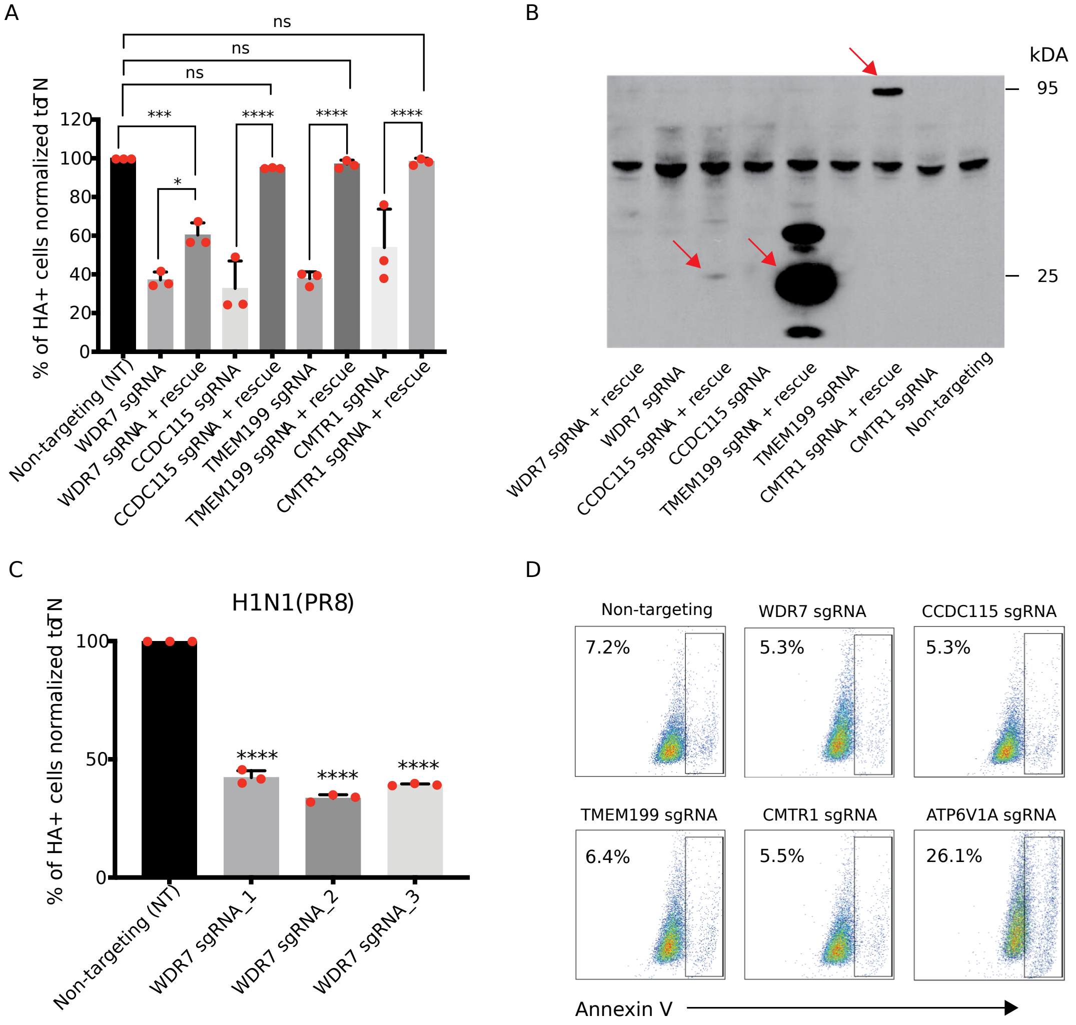

Supplementary Figure 6: Phenotype observed is not due to off-target effects (A) A549 cells we¬re transduced with non-targeting sgRNA, gene-specific sgRNA alone, or gene-specific sgRNA together with a rescue plasmid, followed by infection with PR8 virus at MOI 5 for 16 hours. Y-axis shows percentage of HA-positive cells normalized to non-targeting sgRNA. Error bars represent standard deviations from three independent experimental replicates. (B) A549 cells were transduced with non-targeting sgRNA, gene-specific sgRNA alone, or gene-specific sgRNA together with a rescue plasmid, which expresses a Flag-tagged and codon-shifted version of the targeted gene. The cell lysates were then subjected to SDS-PAGE and western blotting using anti-Flag antibody. Bands representing the proteins of interest were indicated by arrows. Bands were not visible for WDR7 possibly due to large size of the protein (173kDA). (C) A549 cells were transduced with three different sgRNAs targeting WDR7, followed by infection with PR8 virus at MOI 5 for 16 hours. Y-axis shows percentage of HA-positive cells normalized to non-targeting sgRNA. Error bars represent standard deviations from three independent experimental replicates. (D) Annexin V staining of transduced cells on day 9 post-transduction by sgRNA. FACS plots shown are representative of three independent experimental replicates.

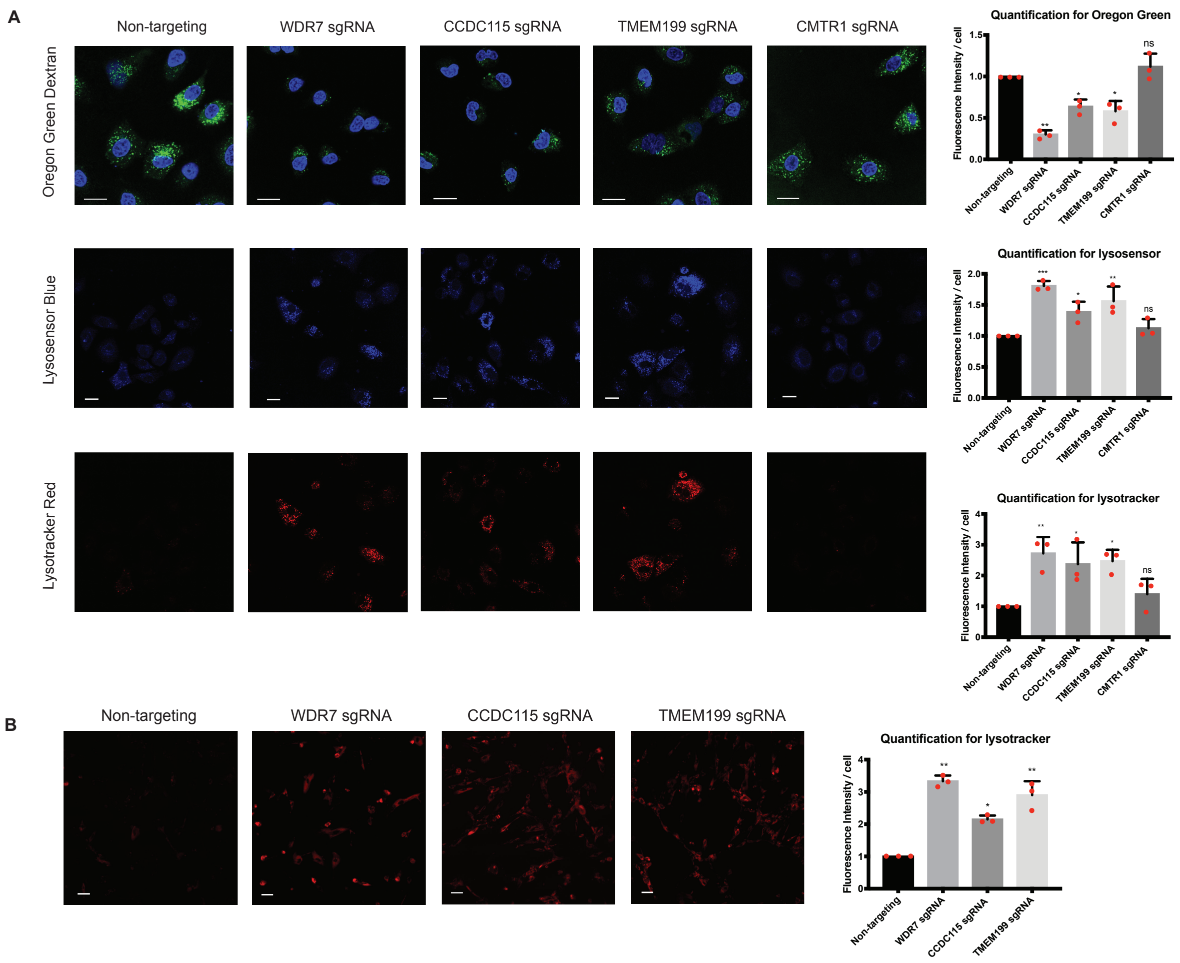

Supplementary Figure 7: WDR7, CCDC115 and TMEM199 regulate endo-lysosome pH. (A) Oregon green dextran (top), Lysosensor blue (middle) and Lysotracker red (bottom) staining of A549 cells transduced with either gene-specific or non-targeting sgRNA. Staining was quantified by dividing total fluorescence intensity by the number of cells in each frame. Bar graphs show the fluorescence intensity/cell in A549 cells transduced with different sgRNAs relative to non-targeting sgRNA. Error bars represent standard deviation from three randomly chosen frames. Scale bar = 20μm. (B) Lysotracker red staining of normal human lung fibroblasts (NHLF) cells transduced with either gene-specific or non-targeting sgRNA. Bar graphs show the fluorescence intensity/cell in NHLF cells transduced with different sgRNAs relative to non-targeting sgRNA.. Error bars represent standard deviation from three randomly chosen frames. Scale bar = 20μm. ***P<0.001 , **P<0.01 and *P<0.05 by one-way ANOVA test.

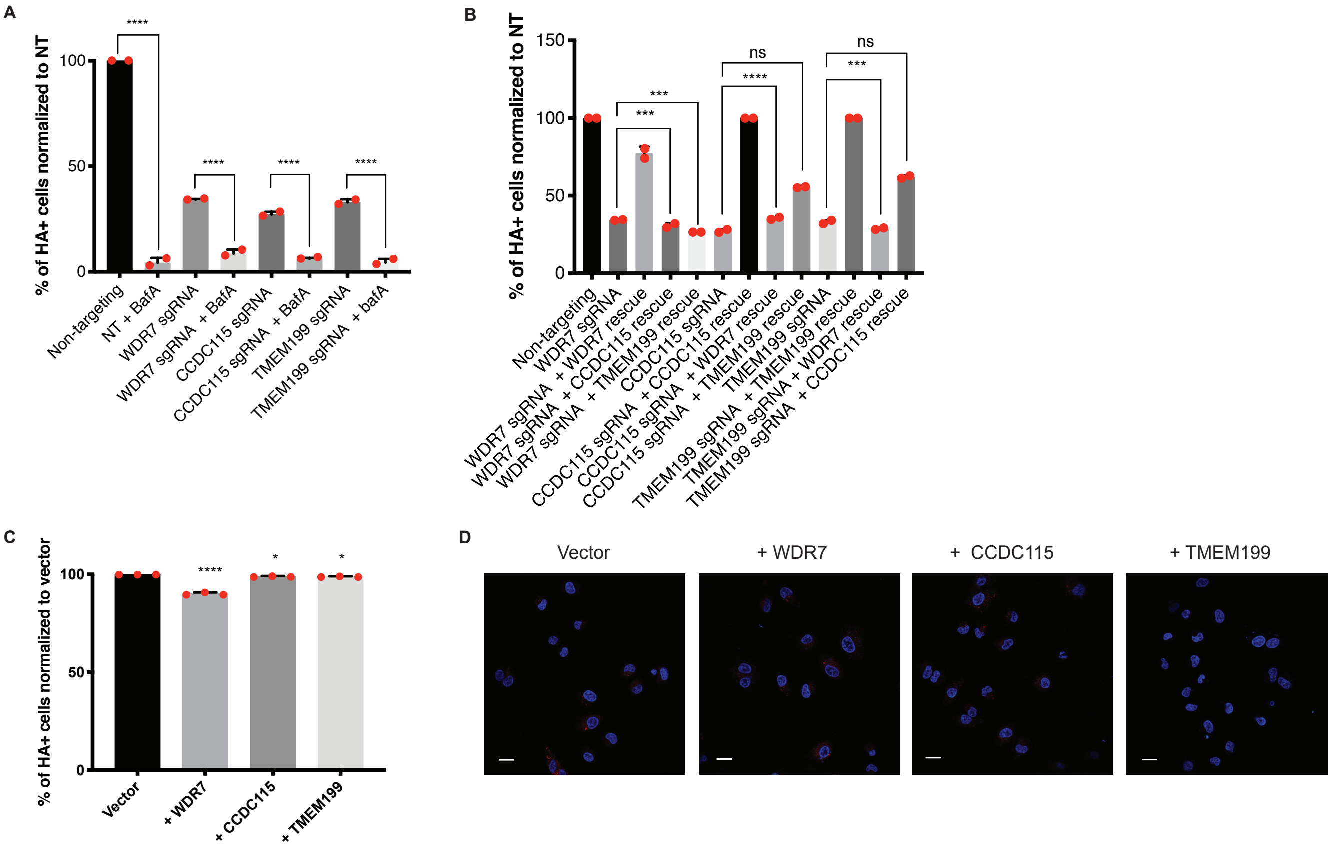

Supplementary Figure 8: WDR7, CCDC115 and TMEM199 play non-redundant roles (A) A549 cells transduced with gene-specific or non-targeting sgRNA were either mock-treated or treated with 100nM BafA for 1 hours at 37°C, followed by PR8 virus infection at MOI 5 for 16 hours. Bar graphs show percentage of HA-positive cells normalized to cells transduced with non-targeting sgRNA. Error bars represent standard deviations from two independent experimental replicates. (B) A549 cells were transduced with either gene-specific sgRNA alone, gene-specific sgRNA with a rescue plasmid expressing a codon-shifted version of the targeted gene, or gene-specific sgRNA with a rescue plasmid expressing a different gene, followed by PR8 virus infection at MOI 5 for 16 hours. Bar graphs show percentage of HA-positive cells normalized to cells transduced with non-targeting sgRNA. Error bars represent standard deviation of two independent experimental replicates. (C) A549 cells were either transduced with lentivirus carrying empty vector or vectors expressing codon-shifted versions of WDR7, CCDC115 or TMEM199, followed by PR8 virus infection at MOI 5 for 16 hours. Bar graphs show percentage of HA-positive cells normalized to cells transduced with lentivirus carrying empty vector. Error bars represent standard deviation of two independent experimental replicates. (D) Lysotracker red staining of A549 cells transduced with lentivirus carrying empty vector or vectors expressing codon-shifted versions of WDR7, CCDC115 or TMEM199. Scale bar = 20μm. ****P=0.0001 ***P<0.001 and *P<0.05, by one-way ANOVA test.

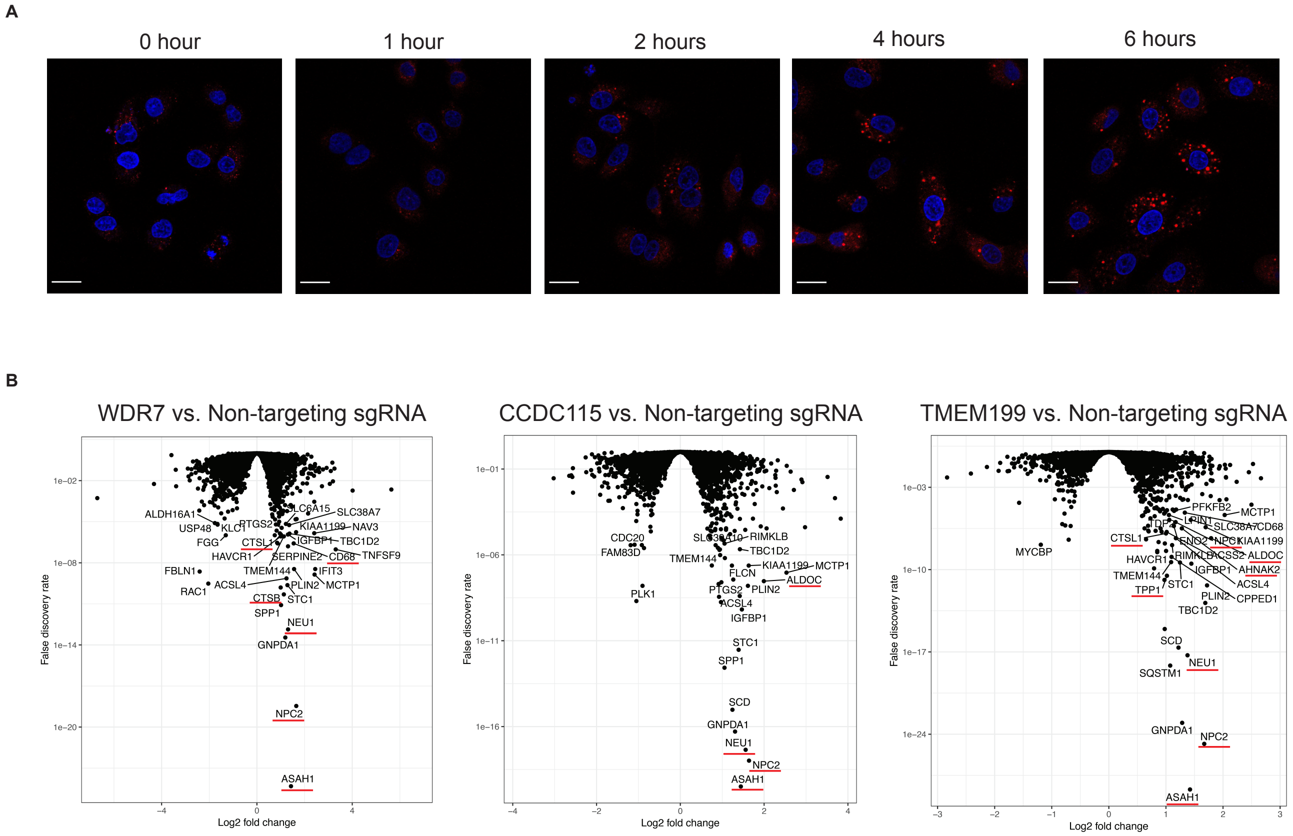

Supplementary Figure 9: WDR7, CCDC115 and TMEM199 regulate lysosomal biogenesis (A) Lysotracker red staining of A549 cells treated with 100µM of Chloroquine at 0, 1, 2, 4 and 6 hours post-treatment. Scale bar = 20μm. (B) Volcano plots showing changes in gene expression between A549 cells transduced with gene-specific and non-targeting sgRNA. False discovery rate (Y-axis) was plotted against Log2 fold change in gene expression (X-axis). Lysosomal genes annotated by the hLGDB database were underlined in red.

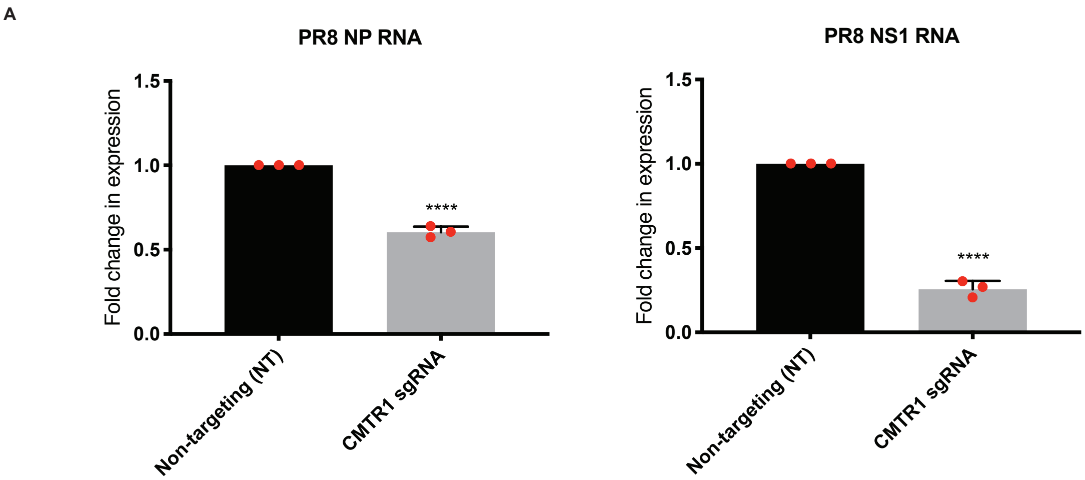

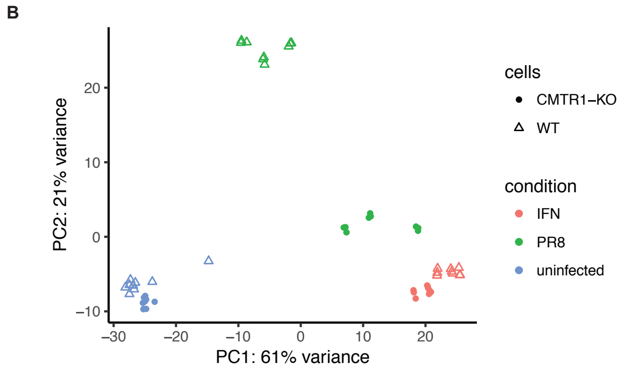

	# up-regulated genes	# down-regulated genes
Uninfected	157	142
PR8	2112	1619
IFN	617	429

Supplementary Figure 10: Loss of CMTR1 leads to increased IFN-β expression. (A) A549 cells were transduced with either CMTR1 or non-targeting sgRNA and infected with PR8 virus at MOI 5 for 16 hours. Fold change in viral NP or NS1 RNA level relative to GAPDH was measured by qRT-PCR and normalized to cells transduced with non-targeting sgRNA. Error bars represent standard deviations from three independent experimental replicates.(B) Principal component analysis (PCA) of RNA sequencing data from wild type and CMTR1 polyclonal KO cells infected with PR8 virus or primed with IFN-β. Each point represents an independent experimental replicate. Table shows number of differentially expressed genes between wild type and CMTR1 KO cells under each condition. Error bars represent standard deviations from three independent experimental replicates. ****P=0.0001, by one-way ANOVA test.

Supplementary References

1.Tripathi, S., Pohl, M.O., Zhou, Y., Rodriguez-Frandsen, A., Wang, G., Stein, D.A., Moulton, H.M., DeJesus, P., Che, J., Mulder, L.C.F., Yángüez, E., Andenmatten, D., Pache, L., Manicassamy, B., Albrecht, R.A., Gonzalez, M.G., Nguyen, Q., Brass, A., Elledge, S., White, M., Shapira, S., Hacohen, N., Karlas, A., Meyer, T.F., Shales, M., Gatorano, A., Johnson, J.R., Jang, G., Johnson, T., Verschueren, E., Sanders, D., Krogan, N., Shaw, M., König, R., Stertz, S., García-Sastre, A. & Chanda, S.K. Meta- and Orthogonal Integration of Influenza "OMICs" Data Defines a Role for UBR4 in Virus Budding. *Cell Host & Microbe* **18**, 723–735(2015).

2.Forrest, A. R. R., Kawaji, H., Rehli, M., Baillie, J.K., et al A promoter-level mammalian expression atlas. *Nature* **507**, 462–470(2014).

3.Han, J., Perez, J.T., Chen, C., Li, Y., Benitez, A., Kandasamy, M., Lee, Y., Andrade, J., tenOever, B. & Manicassamy, B. Genome-wide CRISPR/Cas9 Screen Identifies Host Factors Essential for Influenza Virus Replication. *Cell Reports* **23**, 596–607(2018).

4.König, R., Stertz, S., Zhou, Y., Inoue, A., Hoffmann, H.-H., Bhattacharyya, S., Alamares, J.G., Tscherne, D.M., Ortigoza, M.B., Liang, Y., Gao, Q., Andrews, S.E., Bandyopadhyay, S., De Jesus, P., Tu, B.P., Pache, L., Shih, C., Orth, A., Bonamy, G., Miraglia, L., Ideker, T., García-Sastre, A., Young, J.A.T., Palese, P., Shaw, M.L. & Chanda, S.K. Human host factors required for influenza virus replication. *Nature* **463**, 813–817(2010).

5.Karlas, A., Machuy, N., Shin, Y., Pleissner, K.-P., Artarini, A., Heuer, D., Becker, D., Khalil, H., Ogilvie, L.A., Hess, S., Mäurer, A.P., Müller, E., Wolff, T., Rudel, T. & Meyer, T.F. Genome-wide RNAi screen identifies human host factors crucial for influenza virus replication. *Nature* **463**, 818–822(2010).

6.Brass, A.L., Huang, I.-C., Benita, Y., John, S.P., Krishnan, M.N., Feeley, E.M., Ryan, B.J., Weyer, J.L., Weyden, L. van der, Fikrig, E., Adams, D.J., Xavier, R.J., Farzan, M. & Elledge, S.J. The IFITM proteins mediate cellular resistance to influenza A H1N1 virus, West Nile virus, and dengue virus. *Cell* **139**, 1243–1254(2009).

7.Ward, S.E., Kim, H.S., Komurov, K., Mendiratta, S., Tsai, P.-L., Schmolke, M., Satterly, N., Manicassamy, B., Forst, C.V., Roth, M.G., García-Sastre, A., Blazewska, K.M., McKenna, C.E., Fontoura, B.M. & White, M.A. Host Modulators of H1N1 Cytopathogenicity. *PLoS ONE* **7**, e39284(2012).

8.Su, W.-C., Chen, Y.-C., Tseng, C.-H., Hsu, P.W.-C., Tung, K.-F., Jeng, K.-S. & Lai, M.M.C. Pooled RNAi screen identifies ubiquitin ligase Itch as crucial for influenza A virus release from the endosome during virus entry. *Proceedings of the National Academy of Sciences* **110**, 17516–17521(2013).

9.Tran, A.T., Ranadheera, C., Wilkins, J.A., Cortens, J.P., Opanubi, K.J., Coombs, K.M. & Rahim, M.N. Knockdown of specific host factors protects against influenza virus-induced cell death. *Cell Death & Disease* **4**, e769(2013).

10.Shapira, S.D., Gat-Viks, I., Shum, B.O., Dricot, A., Grace, M.M. de, Wu, L., Gupta, P.B., Hao, T., Silver, S.J., Root, D.E., Hill, D.E., Regev, A. & Hacohen, N. A Physical and Regulatory Map of Host-Influenza Interactions Reveals Pathways in H1N1 Infection. *Cell* **139**, 1255–1267(2009).

11.Watanabe, T., Kawakami, E., Shoemaker, J.E., Lopes, T.J.S., Matsuoka, Y., Tomita, Y., Kozuka-Hata, H., Gorai, T., Kuwahara, T., Takeda, E., Nagata, A., Takano, R., Kiso, M., Yamashita, M., Sakai-Tagawa, Y., Katsura, H., Nonaka, N., Fujii, H., Fujii, K., Sugita, Y., Noda, T., Goto, H., Fukuyama, S., Watanabe, S., Neumann, G., Oyama, M., Kitano, H. & Kawaoka, Y. Influenza Virus-Host Interactome Screen as a Platform for Antiviral Drug Development. *Cell Host & Microbe* **16**, 795–805(2014).

12.Hao, L., Sakurai, A., Watanabe, T., Sorensen, E., Nidom, C.A., Newton, M.A., Ahlquist, P. & Kawaoka,

Y. Drosophila RNAi screen identifies host genes important for influenza virus replication. *Nature* **454**, 890–893(2008).

13.Kanehisa, M. & Goto, S. KEGG: Kyoto encyclopedia of genes and genomes. *Nucleic Acids Research* **28**, 27–30(2000).

14.Matsuoka, Y., Matsumae, H., Katoh, M., Eisfeld, A.J., Neumann, G., Hase, T., Ghosh, S., Shoemaker, J.E., Lopes, T.J., Watanabe, T., Watanabe, S., Fukuyama, S., Kitano, H. & Kawaoka, Y. A comprehensive map of the influenza A virus replication cycle. *BMC Systems Biology* **7**, 97(2013).

15.Carette, J.E., Guimaraes, C.P., Varadarajan, M., Park, A.S., Wuethrich, I., Godarova, A., Kotecki, M., Cochran, B.H., Spooner, E., Ploegh, H.L. & Brummelkamp, T.R. Haploid Genetic Screens in Human Cells Identify Host Factors Used by Pathogens. *Science* **326**, 1231–1235(2009).

16.Sabeti, P.C., Varilly, P., Fry, B., Lohmueller, J., Hostetter, E., Cotsapas, C., Xie, X., Byrne, E.H., McCarroll, S.A., Gaudet, R., Schaffner, S.F., Lander, E.S., Frazer, K.A., Ballinger, D.G., Cox, D.R., Hinds, D.A., Stuve, L.L., Gibbs, R.A., Belmont, J.W., Boudreau, A., Hardenbol, P., Leal, S.M., Pasternak, S., Wheeler, D.A., Willis, T.D., Yu, F., Yang, H., Zeng, C., Gao, Y., Hu, H., Hu, W., Li, C., Lin, W., Liu, S., Pan, H., Tang, X., Wang, J., Wang, W., Yu, J., Zhang, B., Zhang, Q., Zhao, H., Zhao, H., Zhou, J., Gabriel, S.B., Barry, R., Blumenstiel, B., Camargo, A., Defelice, M., Faggart, M., Goyette, M., Gupta, S., Moore, J., Nguyen, H., Onofrio, R.C., Parkin, M., Roy, J., Stahl, E., Winchester, E., Ziaugra, L., Altshuler, D., Shen, Y., Yao, Z., Huang, W., Chu, X., He, Y., Jin, L., Liu, Y., Shen, Y., Sun, W., Wang, H., Wang, Y., Wang, Y., Xiong, X., Xu, L., Waye, M.M.Y., Tsui, S.K.W., Xue, H., Wong, J.T.-F., Galver, L.M., Fan, J.-B., Gunderson, K., Murray, S.S., Oliphant, A.R., Chee, M.S., Montpetit, A., Chagnon, F., Ferretti, V., Leboeuf, M., Olivier, J.-F., Phillips, M.S., Roumy, S., Sallée, C., Verner, A., Hudson, T.J., Kwok, P.-Y., Cai, D., Koboldt, D.C., Miller, R.D., Pawlikowska, L., Taillon-Miller, P., Xiao, M., Tsui, L.-C., Mak, W., Song, Y.Q., Tam, P.K.H., Nakamura, Y., Kawaguchi, T., Kitamoto, T., Morizono, T., Nagashima, A., Ohnishi, Y., Sekine, A., Tanaka, T., Tsunoda, T., Deloukas, P., Bird, C.P., Delgado, M., Dermitzakis, E.T., Gwilliam, R., Hunt, S., Morrison, J., Powell, D., Stranger, B.E., Whittaker, P., Bentley, D.R., Daly, M.J., Bakker, P.I.W. de, Barrett, J., Chretien, Y.R., Maller, J., McCarroll, S., Patterson, N., Pe'er, I., Price, A., Purcell, S., Richter, D.J., Sabeti, P., Saxena, R., Schaffner, S.F., Sham, P.C., Varilly, P., Altshuler, D., Stein, L.D., Krishnan, L., Smith, A.V., Tello-Ruiz, M.K., Thorisson, G.A., Chakravarti, A., Chen, P.E., Cutler, D.J., Kashuk, C.S., Lin, S., Abecasis, G.R., Guan, W., Li, Y., Munro, H.M., Qin, Z.S., Thomas, D.J., McVean, G., Auton, A., Bottolo, L., Cardin, N., Eyheramendy, S., Freeman, C., Marchini, J., Myers, S., Spencer, C., Stephens, M., Donnelly, P., Cardon, L.R., Clarke, G., Evans, D.M., Morris, A.P., Weir, B.S., Tsunoda, T., Johnson, T.A., Mullikin, J.C., Sherry, S.T., Feolo, M., Skol, A., Zhang, H., Zeng, C., Zhao, H., Matsuda, I., Fukushima, Y., Macer, D.R., Suda, E., Rotimi, C.N., Adebamowo, C.A., Ajayi, I., Aniagwu, T., Marshall, P.A., Nkwodimmah, C., Royal, C.D.M., Leppert, M.F., Dixon, M., Peiffer, A., Qiu, R., Kent, A., Kato, K., Niikawa, N., Adewole, I.F., Knoppers, B.M., Foster, M.W., Clayton, E.W., Watkin, J., Gibbs, R.A., Belmont, J.W., Muzny, D., Nazareth, L., Sodergren, E., Weinstock, G.M., Wheeler, D.A., Yakub, I., Gabriel, S.B., Onofrio, R.C., Richter, D.J., Ziaugra, L., Birren, B.W., Daly, M.J., Altshuler, D., Wilson, R.K., Fulton, L.L., Rogers, J., Burton, J., Carter, N.P., Clee, C.M., Griffiths, M., Jones, M.C., McLay, K., Plumb, R.W., Ross, M.T., Sims, S.K., Willey, D.L., Chen, Z., Han, H., Kang, L., Godbout, M., Wallenburg, J.C., L'Archevêque, P., Bellemare, G., Saeki, K., Wang, H., An, D., Fu, H., Li, Q., Wang, Z., Wang, R., Holden, A.L., Brooks, L.D., McEwen, J.E., Guyer, M.S., Wang, V.O., Peterson, J.L., Shi, M., Spiegel, J., Sung, L.M., Zacharia, L.F., Collins, F.S., Kennedy, K., Jamieson, R. & Stewart, J. Genome-wide detection and characterization of positive selection in human populations. *Nature* **449**, 913–918(2007).

17.Ciancanelli, M.J., Abel, L., Zhang, S.-Y. & Casanova, J.-L. Host genetics of severe influenza: From mouse Mx1 to human IRF7. *Current Opinion in Immunology* **38**, 109–120(2016).

18.Horby, P., Nguyen, N.Y., Dunstan, S.J. & Baillie, J.K. An updated systematic review of the role of host genetics in susceptibility to influenza. *Influenza and Other Respiratory Viruses* **7**, 37–41(2013).

19.Chassey, B. de, Aublin-Gex, A., Ruggieri, A., Meyniel-Schicklin, L., Pradezynski, F., Davoust, N., Chantier, T., Tafforeau, L., Mangeot, P.-E., Ciancia, C., Perrin-Cocon, L., Bartenschlager, R., André, P. & Lotteau, V. The Interactomes of Influenza Virus NS1 and NS2 Proteins Identify New Host Factors and Provide Insights for ADAR1 Playing a Supportive Role in Virus Replication. *PLoS Pathog* **9**, e1003440(2013).

20.Hutchinson, E.C., Charles, P.D., Hester, S.S., Thomas, B., Trudgian, D., Martínez-Alonso, M. & Fodor, E. Conserved and host-specific features of influenza virion architecture. *Nature Communications* **5**, (2014).

21.Song, J.-M., Choi, C.-W., Kwon, S.-O., Compans, R.W., Kang, S.-M. & Kim, S.I. Proteomic characterization of influenza H5N1 virus-like particles and their protective immunogenicity. *Journal of Proteome Research* **10**, 3450–3459(2011).

22.Shaw, M.L., Stone, K.L., Colangelo, C.M., Gulcicek, E.E. & Palese, P. Cellular Proteins in Influenza Virus Particles. *PLoS Pathog* **4**, e1000085(2008).

23.Tafforeau, L., Chantier, T., Pradezynski, F., Pellet, J., Mangeot, P.E., Vidalain, P.-O., Andre, P., Rabourdin-Combe, C. & Lotteau, V. Generation and comprehensive analysis of an influenza virus polymerase cellular interaction network. *Journal of virology* **85**, 13010–8(2011).

24.Bradel-Tretheway, B.G., Mattiacio, J.L., Krasnoselsky, A., Stevenson, C., Purdy, D., Dewhurst, S. & Katze, M.G. Comprehensive proteomic analysis of influenza virus polymerase complex reveals a novel association with mitochondrial proteins and rna polymerase accessory factors. *Journal of virology* **85**, 8569–81(2011).

25.Heaton, N.S., Moshkina, N., Fenouil, R., Gardner, T.J., Aguirre, S., Shah, P.S., Zhao, N., Manganaro, L., Hultquist, J.F., Noel, J., Sachs, D., Hamilton, J., Leon, P.E., Chawdury, A., Tripathi, S., Melegari, C., Campisi, L., Hai, R., Metreveli, G., Gamarnik, A.V., García-Sastre, A., Greenbaum, B., Simon, V., Fernandez-Sesma, A., Krogan, N.J., Mulder, L.C.F., Bakel, H. van, Tortorella, D., Taunton, J., Palese, P. & Marazzi, I. Targeting viral proteostasis limits influenza virus, hiv, and dengue virus infection. *Immunity* **44**, 46–58(2016).

26.Jorba, N., Juarez, S., Torreira, E., Gastaminza, P., Zamarreño, N., Albar, J.P. & Ortín, J. Analysis of the interaction of influenza virus polymerase complex with human cell factors. *Proteomics* **8**, 2077–88(2008).

27.Lin, L., Li, Y., Pyo, H.-M., Lu, X., Raman, S.N.T., Liu, Q., Brown, E.G. & Zhou, Y. Identification of rna helicase a as a cellular factor that interacts with influenza a virus ns1 protein and its role in the virus life cycle. *Journal of virology* **86**, 1942–54(2012).

28.Dove, B.K., Surtees, R., Bean, T.J., Munday, D., Wise, H.M., Digard, P., Carroll, M.W., Ajuh, P., Barr, J.N. & Hiscox, J.A. A quantitative proteomic analysis of lung epithelial (A549) cells infected with 2009 pandemic influenza A virus using stable isotope labelling with amino acids in cell culture. *PROTEOMICS* **12**, 1431–1436(2012).

29.Lassmann, T. & Sonnhammer, E.L.L. Automatic assessment of alignment quality. *Nucleic Acids Research* **33**, 7120–7128(2005).
