# Supplementary material for: Genome-wide CRISPR screen identifies host dependency factors for influenza A virus infection
Source: Nat Commun. 2020 Jan 9;11:164. doi: 10.1038/s41467-019-13965-x (PMC6952391; doi:10.1038/s41467-019-13965-x)
Supplement: Supplementary file 2 — Reporting Summary [file 41467_2019_13965_MOESM2_ESM.pdf]

nature research

Corresponding author(s): Nir Hacohen, Ken Baillie

Last updated by author(s): Oct 19, 2019

Reporting Summary

Nature Research wishes to improve the reproducibility of the work that we publish. This form provides structure for consistency and transparency in reporting. For further information on Nature Research policies, see Authors & Referees and the Editorial Policy Checklist.

Statistics

For all statistical analyses, confirm that the following items are present in the figure legend, table legend, main text, or Methods section.

n/a	Confirmed	
☐	☒	The exact sample size (*n*) for each experimental group/condition, given as a discrete number and unit of measurement
☐	☒	A statement on whether measurements were taken from distinct samples or whether the same sample was measured repeatedly
☐	☒	The statistical test(s) used AND whether they are one- or two-sided<br>*Only common tests should be described solely by name; describe more complex techniques in the Methods section.*
☒	☐	A description of all covariates tested
☐	☒	A description of any assumptions or corrections, such as tests of normality and adjustment for multiple comparisons
☐	☒	A full description of the statistical parameters including central tendency (e.g. means) or other basic estimates (e.g. regression coefficient) AND variation (e.g. standard deviation) or associated estimates of uncertainty (e.g. confidence intervals)
☐	☒	For null hypothesis testing, the test statistic (e.g. *F*, *t*, *r*) with confidence intervals, effect sizes, degrees of freedom and *P* value noted<br>*Give P values as exact values whenever suitable.*
☒	☐	For Bayesian analysis, information on the choice of priors and Markov chain Monte Carlo settings
☒	☐	For hierarchical and complex designs, identification of the appropriate level for tests and full reporting of outcomes
☐	☒	Estimates of effect sizes (e.g. Cohen's *d*, Pearson's *r*), indicating how they were calculated

Our web collection on statistics for biologists contains articles on many of the points above.

Software and code

Policy information about availability of computer code

Data collection	Data analyses as specified in the methods section were performed using python 3.7 including pandas 0.23.4 and numpy 1.14.5. Quantile normalisation was performed using biocLite in R 3.5.0.
Data analysis	A repository of the software developed for this project can be downloaded here https://github.com/baillielab/maic

For manuscripts utilizing custom algorithms or software that are central to the research but not yet described in published literature, software must be made available to editors/reviewers. We strongly encourage code deposition in a community repository (e.g. GitHub). See the Nature Research guidelines for submitting code & software for further information.

Data

Policy information about availability of data

All manuscripts must include a data availability statement. This statement should provide the following information, where applicable:

- Accession codes, unique identifiers, or web links for publicly available datasets
- A list of figures that have associated raw data
- A description of any restrictions on data availability

The authors declare that all data supporting the findings of this study are available within the paper and its supplementary information files.

Field-specific reporting

Please select the one below that is the best fit for your research. If you are not sure, read the appropriate sections before making your selection.

☒ Life sciences ☐ Behavioural & social sciences ☐ Ecological, evolutionary & environmental sciences

Life sciences study design

All studies must disclose on these points even when the disclosure is negative.

Sample size	For CRISPR/Cas9 screen, 80 million cells were transduced with the sgRNA library to achieve a minimum coverage of 1000x per sgRNA. The cells were then expanded for 8 days and approximately 300 million cells were infected with Influenza A PR8 virus. For validation of target genes, 0.2 million cells were infected with PR8 virus followed by either FACS staining or plaque assay. For confocal microscopy, 50,000 cells were seeded on chambered cover-glass prior to staining.
Data exclusions	No data were excluded from analyses.
Replication	Experiments were performed in three biological replicates. All attempts for replication were successful for data included in the manuscript.
Randomization	Randomization was not relevant to the current study. All experiments were performed on cell lines and no human/animal cohort was involved.
Blinding	Blinding was done for experiments with higher tendency for biases and those performed by collaborators. These included experiments involving microscopy images, plaque assay counting and anti-eIF4E immuno-precipitation.

Reporting for specific materials, systems and methods

We require information from authors about some types of materials, experimental systems and methods used in many studies. Here, indicate whether each material, system or method listed is relevant to your study. If you are not sure if a list item applies to your research, read the appropriate section before selecting a response.

Materials & experimental systems

n/a	Involved in the study	
☐	☒	Antibodies
☐	☒	Eukaryotic cell lines
☒	☐	Palaeontology
☒	☐	Animals and other organisms
☒	☐	Human research participants
☒	☐	Clinical data

Methods

n/a	Involved in the study	
☒	☐	ChIP-seq
☐	☒	Flow cytometry
☒	☐	MRI-based neuroimaging

Antibodies

Antibodies used	From EMD Millipore, Anti-Influenza A HA (AB1074), FITC Anti-Influenza A Nucleoprotein clone A1 (MAB8257F). From Abcam, Anti-LAMP1 clone H4A3 (ab25630), Anti-Rab7 Alexa-Fluor647 clone EPR7589 (ab198337), β-actin antibody (ab6276). From BD bioscience, FITC mouse anti-human CD71 (555536). From Thermofisher, Alexa-Fluor488 Goat anti-mouse IgG, Alexa-Fluor488 Donkey anti-goat IgG. From Sigma Aldrich, Anti-Flag M2 antibody (F3165). From Cell Signaling Technology, TFEB antibody (#4240S), Phospho-TFEB antibody (Ser211) (37681S).
Validation	All antibodies used in this study have been cited in previous publications. These could be found on the manufacturers' websites.

Eukaryotic cell lines

Policy information about cell lines

Cell line source(s)	A549 cells, HEK293T cells, MDCK cells and VERO cells were obtained from ATCC. Primary human lung fibroblast cells were obtained from Lonza.
Authentication	Cell lines were not authenticated.
Mycoplasma contamination	All cell lines were tested negative for Mycoplasma.
Commonly misidentified lines (See ICLAC register)	No commonly misidentified lines were used.

Flow Cytometry

Plots

Confirm that:

☒ The axis labels state the marker and fluorochrome used (e.g. CD4-FITC).

☒ The axis scales are clearly visible. Include numbers along axes only for bottom left plot of group (a 'group' is an analysis of identical markers).

☒ All plots are contour plots with outliers or pseudocolor plots.

☒ A numerical value for number of cells or percentage (with statistics) is provided.

Methodology

Sample preparation	Harvested cells were washed with ice cold PBS and stained with primary antibodies in FACS buffer (PBS + 1% BSA) for 30 minutes. Cells were then washed twice with ice cold PBS and stained with secondary antibody on ice for 30 minutes, followed by fixation with 4% paraformaldehyde. For intracellular staining for Influenza A nucleoprotein (NP), cells were fixed and permeabilized using 0.1% Saponin (Sigma Aldrich) prior to antibody staining. No tissue preparation was required as all cells used were from cell lines.
Instrument	FACSAria II was used for cell sorting and Accuri C6 was used for analyses. Both instruments are from Bd Bioscience.
Software	FACSDiva was used for cell sorting and Accuri C6 software was used for data acquisition and analysis on the instruments. Flowjo software (version 10) was used for analyses on personal computers.
Cell population abundance	For the CRISPR/Cas9 screen, approximately 2-5 million cells were sorted into the uninfected bin and 40 million cells were sorted into the control bin. Cell purity in both bins were around 90-95% and was determined by re-running the sorted samples on the FACSAria II.
Gating strategy	For the CRISPR/Cas9 screen, cells were stained with anti-Influenza HA primary antibody and Alexa-Fluo488 secondary antibody. Cells with the lowest HA staining in the normal distribution (5% of the total population) were sorted into the uninfected bin. Cells with moderate HA staining (+ - 20% of the modal) were sorted into the control bin. For validation studies, FSC/SSC gating was only used to distinguish between live cells and debris since cell lines were used. Cells that are infected or uninfected with Influenza virus show a bimodal distribution of surface HA expression.

☐ Tick this box to confirm that a figure exemplifying the gating strategy is provided in the Supplementary Information.
