# Supplementary material for: Genome-wide CRISPR screen identifies host dependency factors for influenza A virus infection
Source: Nat Commun. 2020 Jan 9;11:164. doi: 10.1038/s41467-019-13965-x (PMC6952391; doi:10.1038/s41467-019-13965-x)
Supplement: Supplementary file 3 — Description of Additional Supplementary Files [file 41467_2019_13965_MOESM3_ESM.pdf]

Description of Additional Supplementary Files

File Name: Supplementary Data 1

Description: **Ranked list of hits from CRISPR/Cas9 screens** Genes are ranked by ascending order of their false discovery rate (FDR). For the individual columns, **screen p** is the p-value of hits from the primary screens. **Validation p** is the p-value of hits from the secondary screen. **Combined p** is the p-value of hits combining results from the primary and secondary screens. **Combined fdr** is the FDR of hits calculated from the combined p values**. Screen remainder p** is the p-value of hits after leaving out the strongest effect sgRNA. **Multiple sgRNA support** indicates if a particular hit shows enrichment in two or more sgRNAs.

File Name: Supplementary Data 2

Description: **Ranked list of genes from Meta-Analysis by Information Content (MAIC)** Genes are ranked by descending order of their total MAIC scores, which is derived from the sum of MAIC scores of all input data source **(refer to experimental procedures).** Genes with higher total MAIC scores are better supported by pre-existing evidence to be important for IAV infection. For the individual columns, **CRISPR p** is the p-value of hits from the primary screens. **Validation p** is the p-value of hits from the secondary screen. **Combined p** is the p-value of hits combining results from the primary and secondary screens. **FDR** of the hits are calculated from the Combined p values. MAIC scores are reported for 9 different input data source, including **CRISPR, RNAi screens, Protein interaction, Proteomics, Haploid Screens, Positive Selection, Database Annotation, Gene Expression and Genetics.** A gene is included in the core host set if its total MAIC score is greater than 2 standard deviations above mean and is non-zero in either RNAi or CRISPR. Table shows ranked list of genes with and without ribosomal genes. Gene set enrichment analysis (GSEA) was performed for the MAIC ranked genes using the Biocarta and KEGG gene set libraries to highlight important pathways involved in IAV infection.

File Name: Supplementary Data 3

Description: **Overlap in hits between our screen and previous studies** Table showing simple overlap in hits between our screen and previous RNAi screens, proteomics studies and CRISPR screen. Genes are ranked alphabetically.
